# White collar 1‐induced photolyase expression contributes to UV‐tolerance of *Ustilago maydis*

**DOI:** 10.1002/mbo3.322

**Published:** 2015-12-20

**Authors:** Annika Brych, Judita Mascarenhas, Elaine Jaeger, Elzbieta Charkiewicz, Richard Pokorny, Michael Bölker, Gunther Doehlemann, Alfred Batschauer

**Affiliations:** ^1^ Faculty of BiologyDepartment of Plant Physiology and PhotobiologyPhilipps‐UniversityKarl‐von‐Frisch‐Str. 8Marburg35032Germany; ^2^ Faculty of BiologyDepartment of GeneticsPhilipps‐UniversityKarl‐von‐Frisch‐Str. 8Marburg35032Germany; ^3^ Department of Organismic InteractionsMax‐Planck‐Institute for Terrestrial MicrobiologyKarl‐von‐Frisch‐Str. 10Marburg35043Germany; ^4^ Present address: MPI für terrestrische MikrobiologieLOEWE‐Zentrum für Synthetische Mikrobiologie (SYNMIKRO)Karl‐von‐Frisch‐Straße 16Marburg35043Germany; ^5^ Present address: Botanical Institute and Cluster of Excellence on Plant Sciences (CEPLAS)University of CologneCologne50674Germany

**Keywords:** DNA‐repair, light regulation, photolyase, *U. maydis*, UV‐tolerance, white collar 1

## Abstract

*Ustilago maydis* is a phytopathogenic fungus causing corn smut disease. It also is known for its extreme tolerance to UV‐ and ionizing radiation. It has not been elucidated whether light‐sensing proteins, and in particular photolyases play a role in its UV‐tolerance. Based on homology analysis, *U. maydis* has 10 genes encoding putative light‐responsive proteins. Four amongst these belong to the cryptochrome/photolyase family (CPF) and one represents a white collar 1 ortholog (*wco1*). Deletion mutants in the predicted cyclobutane pyrimidine dimer CPD‐ and (6–4)‐photolyase were impaired in photoreactivation. In line with this, in vitro studies with recombinant CPF proteins demonstrated binding of the catalytic FAD cofactor, its photoreduction to fully reduced FADH ^−^ and repair activity for cyclobutane pyrimidine dimers (CPDs) or (6–4)‐photoproducts, respectively. We also investigated the role of Wco1. Strikingly, transcriptional profiling showed 61 genes differentially expressed upon blue light exposure of wild‐type, but only eight genes in the *Δwco1* mutant. These results demonstrate that Wco1 is a functional blue light photoreceptor in *U. maydis* regulating expression of several genes including both photolyases. Finally, we show that the *Δwco1* mutant is less tolerant against UV‐B due to its incapability to induce photolyase expression.

## Introduction

Light is one of the most common stimuli used by living organisms to interact with the environment. Photoreceptors absorb the light, get activated, and relay the molecular signal downstream to trigger complex developmental and other responses (Briggs and Spudich 2005).

Filamentous fungi possess a wide variety of photoreceptors. During their life cycle, they are exposed to various habitats which differ in the availability of nutrients and water, temperature, and oxygen. These changes are also reflected in their light environment (fluence rates, spectral composition) (Rodriguez‐Romero et al. 2010; Fuller et al. 2015). One common aspect of light‐regulated fungal development is reproduction. As sessile organisms, they need to disperse their spores outside the original substrate in order to tap new resources and favorable habitats. In addition, exposure to sunlight also causes formation of reactive oxygen species and other photoproducts such as DNA lesions (Cadet and Wagner 2013). Accordingly, most fungi show light‐induced expression of defense genes which protect against or repair such lesions, and formation of pigments which filter visible light and/or UV (Braga et al. 2015).

The recent progress in identifying fungal photoreceptors and elucidating their biological functions is tremendous and reviewed in several articles (Avalos and Estrada 2010; Bayram et al. 2010; Braus et al. 2010; Chen et al. 2010; Corrochano and Garre 2010; Idnurm et al. 2010; Kamada et al. 2010; Rodriguez‐Romero et al. 2010; Schmoll et al. 2010; Fuller et al. 2015). The photobiology and the role of specific photoreceptors have been so far investigated in detail in *Neurospora crassa*,* Trichoderma* sp., *Aspergillus nidulans*,* Fusarium* sp., *Magnaporthe oryzae* (Ascomycota); *Cryptococcus neoformans*,* Coprinopsis cinerea* (Basidomycota); *Phycomyces blakesleeanus*, and *Mucor circinelloides* (Mucormycotina). In contrast, very little is known about the photobiology of *U. maydis* albeit its genome encodes all photoreceptors so far identified in other fungi. In addition, it encodes a blue light sensing using flavin (BLUF)‐domain protein which it shares only with closely related species.

In this study, we focus on blue light responses in *U. maydis* and investigate the role of orthologous proteins known to be involved in blue light perception in other fungal systems. Among them is the white collar complex (WCC), a protein complex formed of White Collar 1 (Wco1) and White Collar 2 (Wco2). WCC was originally identified in *N. crassa* based on screens for blue light‐insensitive mutants (Degli‐Innocenti et al. 1984; Nelson et al. 1989; Ballario and Macino 1997; Linden et al. 1997). WC‐1 from *N. crassa* (Ballario et al. 1996) is organized in an N‐terminal receiver domain, which contains three PAS (PER/ARNT/SIM) domains and a zinc‐finger domain located at the C‐terminus. One of the PAS domains belongs to a subgroup of PAS domains, which were assigned as LOV (light/oxygen/voltage) domain and bind the flavin chromophore (Huala et al. 1997). For WC‐1, FAD has been identified as chromophore (Froehlich et al. 2002; He et al. 2002). LOV(light/oxygen/voltage)‐domain photoreceptors perceive UV‐A/blue light and undergo a photocycle with a transiently formed flavin adduct at a conserved cysteine residue in the LOV‐domain (reviewed in Swartz and Bogomolni 2005). Like *wc‐1*, the *wc‐2* gene was first characterized in *N. crassa* (Linden and Macino 1997). WC‐2 has a PAS‐domain and a zinc‐finger domain, and heterodimerizes with WC‐1 to form a light‐responsive transcription factor (Froehlich et al. 2002; He et al. 2002) that binds to so called early light‐responsive elements (Froehlich et al. 2002; He et al. 2002; Kaldi et al. 2006). Genes endowed with these elements (class I genes) show typically a very fast and transient induction with peaks between 30–60 min after light onset, followed by class II and class III genes. In contrast to class I and II genes, class III genes show no light adaptation (Shrode et al. 2001; Lewis et al. 2002; Schwerdtfeger and Linden 2003). In *N. crassa,* transcriptomic studies revealed up to 6% of genes controlled by light (Chen et al. 2009). White collar complex here plays a dominant role. Despite its clear photoreceptor function, WCC also has important roles which are independent of light, they regulate the circadian feedback loop in *N. crassa* (Crosthwaite et al. 1997), or virulence of *C. neoformans* in mammalian hosts (Idnurm and Heitman 2005).

*wc‐1* and *wc‐2* genes are present in essentially all Mycota with some exceptions such as *Saccharomyces cerevisiae*, and some species of Hemiascomycota, Archiascomycota, and Zygomycota (Idnurm et al. 2010; Rodriguez‐Romero et al. 2010). Analysis of *wc‐1* mutants in many filamentous fungal species revealed several light responses mediated by WC‐1 including conidiation, conidia release, mycotoxin biosynthesis, inhibition of mating, increased UV‐tolerance, carotenogenesis, clock entrainment, and phototropism (reviewed in Idnurm et al. 2010). In contrast to Ascomycetes, *wc‐1* genes from Basidiomycetes lack a zinc‐finger domain (Idnurm and Heitman 2005).

Members of the cryptochrome/photolyase family (CPF) are present in all kingdoms of life (Chaves et al. 2011). Photolyases (PHR) repair the two major UV‐B lesions in DNA, namely cyclobutane pyrimidine dimers (CPDs) and (6–4) photoproducts. Each lesion is repaired by a specific class of photolyase (CPD‐photolyase or (6–4)‐photolyase). Cryptochromes (CRY) have photoreceptor function or are integral components of the circadian clock as in mammals. Both, photolyases and cryptochromes carry FAD as essential cofactor/chromophore. For most of them, a second chromophore such as methenyltetrahydrofolate (MTHF) was identified, which functions as antenna and transfers excitation energy to FAD. The protein structures of several photolyases and a few cryptochromes have been solved and show striking similarity in their overall fold (Essen 2006; Müller and Carell 2009; Kiontke et al. 2011). A more recently characterized subgroup of the CPF consists of cry‐DASH proteins. Originally, they were considered as photoreceptors (Brudler et al. 2003; Kleine et al. 2003), but later studies showed that they repair CPD lesions in single‐stranded DNA (Selby and Sancar 2006) and loop structures of double‐stranded DNA (Pokorny et al. 2008). In filamentous fungi CPD‐photolyases, (6–4)‐photolyases as well as cry‐DASH proteins were identified (Bayram et al. 2008a; Avalos and Estrada 2010; Idnurm et al. 2010). Analysis of some photolyases including fungal ones showed that photolyases may have a dual function as repair enzyme and photoreceptor. The *A. nidulans cryA* gene groups with class I CPD‐photolyase. However, its deletion mutant showed increased sexual fruiting body formation and conidiation under UV‐A and blue light, respectively, which corresponded to increase in the levels of genes that regulate sexual development (Bayram et al. 2008a; Avalos and Estrada 2010). Thus, CryA is a *bona fide* UV‐A/blue light photoreceptor. Other examples of dual function enzymes/receptors from fungi are: Class I CPD‐photolyase of *Trichoderma atroviride*, that regulates the photoinduction of its own gene (Berrocal‐Tito et al. 2007); DASH‐type cry of *N. crassa* that acts as a regulator of one circadian oscillator in this fungus (Nsa et al. 2015), and its deficiency has some effect on light entrainment of the circadian clock and results in the light‐dependent upregulation of few genes including VIVID (*vvd*) (Froehlich et al. 2010; Olmedo et al. 2010); DASH‐type cry in *S. sclerotiorum* and *F. fujikuroi* (Veluchamy and Rollins 2008; Castrillo et al. 2013); and the predicted (6–4)‐photolyase from *Cercospora zeae‐maydis* which induces the CPD‐photolyase and other genes involved in DNA repair (Bluhm and Dunkle 2008).

*Ustilago maydis* is a basidiomycete plant pathogen that infects *Zea mays* causing corn smut disease (Christensen 1963). Its life cycle consists of a haploid, nonpathogenic, saprophytic phase in soil, and a dikaryotic, biotrophic phase in above‐ground organs of the plant (Kahmann et al. 2000; Djamei and Kahmann 2012). *U. maydis* is not only an excellent model organism to study plant–pathogen interaction, but serves likewise for eukaryotic genetics, cell biology and signaling (Banuett 1995; Brefort et al. 2009; Vollmeister et al. 2011). Its genome is completely sequenced (Kämper et al. 2006) and several molecular techniques are well established (Kämper 2004; Steinberg and Perez‐Martin 2008; Heimel et al. 2010; Schuster et al. 2011).

Having a life cycle with a saprophytic and biotrophic phase and thus living in different habitats, *U. maydis* also is an excellent model organism to study response and adaption to various environmental cues. Surprisingly, little is known about its photobiology and the role of specific photoreceptors in overall growth and responses to the environment. In this study, we explore the blue light response and characterize some of the photoreceptors in *U. maydis*. We have identified several putative photoreceptors for the UV‐A/blue region including members of the CPF and a *wc‐1* ortholog (*wco1*). *U. maydis* responds to blue light by regulating a battery of genes controlled by Wco1, among them are the photolyases. Biochemical characterization of the CPF proteins confirmed their role in DNA repair and UV‐tolerance of *U. maydis*.

## Materials and Methods

### *U. maydis* strains, media, and culture conditions

*U. maydis* strains used in this study are listed in Table S1. Strain FB1 (Banuett and Herskowitz 1989) served as the wild‐type. Cells were grown at 28–30°C shaking at 200 rpm in YEPS‐L (0.4% yeast extract, 0.4% peptone and 2% sucrose; Tsukuda et al. 1988) or YNB‐SO_4_ medium with 2% glucose (Mahlert et al. 2006; Freitag et al. 2011) or on agar plates with potato dextrose (PD). Data presented for *U. maydis* mutants are based on analyses of at least three independent lines.

### Construction of *U. maydis* strains

Standard molecular techniques were used (Sambrook et al. 1989). All enzymes if not otherwise stated were purchased from New England Biolabs (Frankfurt/Main, Germany). Isolation of *U. maydis* genomic DNA was carried out according to a published protocol (Hoffman and Winston 1987). All *U. maydis* strains generated in this study are derived from the wild‐type isolate FB1. For the deletion of *wco1* (*um03180*)*, cry1* (*um01131*), *cry2* (*um05917*), *phr1* (*um06079*), and *phr2* (*um02144*), a PCR‐based approach using hygromycin as resistance marker (Kämper 2004) was applied. 1 kb of each flanking region of each gene was amplified by PCR using primers for the left border and primers for the right border (Table S2). PCR products were digested with *Sfi*I and ligated to the hygromycin cassette of pMF1‐h (Brachmann et al. 2004).

All mutant strains were confirmed by PCR and Southern analysis. Deletion phenotypes were verified by complementation. Transformation of *U. maydis* was performed as described (Tsukuda et al. 1988; Schulz et al. 1990). For selection of transformants, PD plates containing 200 *μ*g mL^−1^ hygromycin or 5 *μ*g mL^−1^ carboxin were used.

For construction of *U. maydis* strains expressing GFP‐Wco1 or mCherry‐Wco2 the *wco1* (*um03180*) or *wco2* (*um02664*), open reading frames were cloned downstream of the fluorescent protein in a plasmid derived from Böhmer et al. (2008). Primers used are listed in Table S3. The protein fusion was expressed from the *otef* promoter. Plasmids were linearized with *Ssp*I and integrated into the *ip* locus of FB1. The integration of the plasmids into the ectopic *ip* locus and expression of the fusion proteins were verified by PCR and subsequently by immunoblotting (Brachmann et al. 2001).

### Light treatments of *U. maydis*

Cells were grown in YEPS‐L medium at 28°C and dark‐adapted by incubation overnight in complete darkness. The following day cultures were diluted in YNB‐SO_4_ medium with 2% glucose under green safe light to an OD_600_ of 0.2 followed by incubation for 3 h in darkness. Cultures were then split in two aliquots. One aliquot, the dark sample, was completely wrapped with aluminum foil and kept with the other uncovered aliquot on the same shaker during the blue light (471 nm, 30 *μ*mol m^−2^ sec^−1^) treatment for 60 min. Cells were pelleted and stored at −80°C until RNA extraction.

### Photoreactivation tests

To test for photoreactivation after UV‐B treatment, wild‐type and mutant cells were grown until logarithmic phase in YEPS‐L. The cultures were diluted to an OD_600_ of 0.1 and 50 *μ*L of various dilutions (1:100, 1:300, and 1:900) were spread on PD‐plates with some distance to the border to avoid shadowing of the cells by the rim of the plates. Plates were irradiated for 0, 40, 80, 120 or 180 sec with UV‐B (1.83 W m^−2^; for spectrum see Fig. S1) from seven tubes (Ultraviolet‐B TL 40W/12 RS; Philips, Amstelplein, Netherlands) and incubated afterwards in darkness or illuminated for 1 h with white light (OSRAM 36W/11 TL LUMILUX daylight, distance between plates and light field: 43 cm) followed by incubation in the dark for 48 h. Colonies were counted to determine the survival rate.

### RNA isolation, qRT‐PCR, and microarray analysis

RNA was extracted using TRIzol Reagent (Life technologies, Darmstadt, Germany). For microarrays, 6 *μ*g RNA were treated with 1 *μ*L Precision DNase (Primerdesign, Rownhams, UK) and purified using an RNeasy Mini Kit (Qiagen, Hilden, Germany). Labeling of 100 ng RNA and hybridization on custom‐designed Affymetrix chips (Eichhorn et al. 2006) was achieved with the GeneChip 3′ IVT PLUS Reagent Kit (Affymetrix, Cologne, Germany) using the protocol FS450_0001 at the GeneChip Fluidics Station and the instructions of the Affymetrix GeneChip Command Console for the GeneArray Scanner.

The microarray data were analyzed using the Partek Genomics Suite version 6.12. Expression values were normalized using the RMA method. Criteria for significance were a corrected *P*‐value (per sample) with an FDR of 0.05 and a fold change of >2. Differentially expressed genes were calculated by a one‐way ANOVA. Array data are based on three biological replicates.

To verify microarray results, selected genes were analyzed by quantitative RT‐PCR. Therefore, 2 *μ*g of RNA were treated with 1 *μ*L of Precision DNase, and then, cDNA was synthesized using the Precision nanoScript Reverse Transcription Kit (both from Primerdesign, Rownhams, UK). Quantitative RT‐PCR was performed on a Rotor‐GeneQ cycler (Qiagen, Hilden, Germany) using the SensiFast SYBR No‐ROX‐Kit (Bioline, Luckenwalde, Germany). Cycling conditions were 3 min 95°C, followed by 45 cycles of 5 sec 95°C, 10 sec 60°C, 30 sec 72°C, and an increase in temperature from 72°C to 95°C for melting analysis.

### Yeast‐two‐hybrid studies

The Matchmaker two‐hybrid system from Clontech (BD Biosciences Clontech, Palo Alto, CA) was used to study *U. maydis* White collar protein interactions. The *Saccharomyces cerevisiae* strain Y190 was cotransformed with pAS2‐ and pACT2‐ derived plasmids. pAS2 was used to express Wco1 or Wco2 as fusions to DNA binding domain (BD), and Wco1 or Wco2 were expressed as fusions to the activation domain (AD) of GAL4 in pACT2. Double transformants were selected on minimal medium lacking leucine and tryptophan (‐L, ‐W), and subsequent spotting of cell suspensions on the same medium (as control) or minimal medium lacking leucine, tryptophan, and histidine and containing 3AT (3‐amino‐1,2,4‐triazole). The protein interactions were determined by growth on selective medium. The activation of ß‐galactosidase was tested with X‐gal as substrate by a filter lift assay (Möckli and Auerbach 2004).

### Fluorescence microscopy

*Ustilago maydis* cells from logarithmic phase grown in YNB‐SO_4_ medium with 2% glucose were mounted on agarose padded slides. Fluorescence microscopy was performed on a Zeiss Axiovert 200 microscope system using a CCD camera. Image acquisition was performed using Improvision Volocity software and processed on ImageJ. Staining of nuclei with Hoechst 33,342 dye was done as described before (Kangatharalingam and Ferguson 1984).

### Construction of *E. coli* expression vectors and expression of recombinant proteins

The synthetic cDNA encoding *U. maydis cry1* (Life Technologies) was cloned using KpnI and SacI sites into pET51b expression vector (Novagen/Merck, Darmstadt, Germany) and expressed as 10xHis‐ and Strep‐tagged protein in *E.coli* Bl21 (DE3) cells (Table S4). Cells were grown in autoinduction medium (0.5% yeast extract, 1% tryptone, 1 mmol L^−1^ MgSO_4_, 25 mmol L^−1^ (NH_4_)_2_SO_4_, 50 mmol L^−1^ KH_2_PO_4_, 50 mmol L^−1^ Na_2_HPO_4_, 0.5% glycerol, 0.05% glucose, 0.2% *α*‐lactose) at 20°C for 40 h.

The cDNA encoding *U. maydis cry2* was cloned using SalI and NotI sites into pET51b expression vector and expressed as 10xHis‐ and Strep‐tagged protein in *E. coli* Arctic Express cells. Cells were grown in LB (lysogeny broth) medium (0.5% yeast extract, 1% tryptone, 1% NaCl). Protein expression was induced by the addition of 1 mmol L^−1^ IPTG and cells were further incubated for 16 h at 16°C.

The cDNA encoding *U. maydis phr2* was cloned using KpnI and NotI sites into pET51b expression vector and expressed as 10xHis‐ and Strep‐tagged protein in *E. coli* Rosetta (DE3) cells. Expression was as for Cry1.

The cDNA encoding *U. maydis phr1* was cloned using NcoI and NotI sites into a modified pET21d expression vector (Hothorn et al. 2011) and expressed as 7xHis‐ and Strep‐tagged SUMO fusion protein in *E.coli* BL21 (DE3) cells. Cells were grown in TB (Terrific Broth) medium (2.4% yeast extract, 1.2% tryptone, 0.4% glycerol, 17 mmol L^−1^ KH_2_PO_4_, 72 mmol L^−1^ K_2_HPO_4_) at 25°C for 20 h. Primers for cloning of CPF members in expression vectors are listed in Table S5.

The same procedure of cells harvesting, cell lysis, and purification of recombinant CPF proteins was applied as described previously (Pokorny et al. 2005). In brief, the protein was purified by two chromatographic steps. For the first step, Ni^+2^‐affinity chromatograph (GE Healthcare, Munich, Germany) was used. The proteins were eluted by a linear gradient of imidazole from 10 to 500 mmol L^−1^ For the second heparin HiTrap column purification step (GE Healthcare, Munich, Germany), the protein was eluted by a linear gradient of NaCl from 0.2 to 2 mol L^−1^.

### Photoreduction of CPF proteins

10 *μ*mol L^−1^ of proteins were illuminated with blue light (Cry1: 450 nm, 10 nm FWHM, 50 *μ*mol m^−2^ sec^−1^; Cry2: 450 nm, 10 nm FWHM, 64 *μ*mol m^−2^ sec^−1^; Phr2: 439 nm, 10 nm FWHM, 38 *μ*mol m^−2^ sec^−1^; Phr1: 450 nm, 10 nm FWHM, 100 *μ*mol m^−2^ sec^−1^) in the presence of 10 mmol L^−1^ DTT at 15°C for 30, 45, or 60 min. The final buffer conditions were 50 mmol L^−1^ Na‐phosphate pH 7.5, 200 mmol L^−1^ NaCl, 10% glycerol. During the blue light illumination, absorption changes were monitored using UV‐Vis spectrophotometer (UV‐240 1 PC; Shimadzu, Neufahrn, Germany).

### DNA repair assays

For the photorepair of T<>T dimers in dsDNA by Phr1, a restriction site restoration assay was used as described before (Pokorny et al. 2008). The reaction contained 40 nmol L^−1^ Phr1, 2 nmol L^−1^ oligoLAMRA (for sequence see Table S6), 2 mmol L^−1^ DTT, 10% 10 × buffer O (Fermentas/Fisher Scientific, Schwerte, Germany), 10% glycerol. Samples were placed in a Quartz Suprasil cell (Hellma GmbH & Co.KG, Müllheim, Germany) and irradiated with UV‐A (365 nm, 88 *μ*mol m^−2^ sec^−1^) at 15°C. The control was stored in darkness at 15°C. After the blue light treatment, the samples were incubated at 95°C for 10 min to inactivate Phr1 and annealed with oligoCT (40‐mer fully complementary strand to oligoLAMRA; for sequence see Table S6). In the next step, samples were incubated with *Vsp*I (Fermentas/Fisher Scientific) at 37°C for 60 min, afterwards *Vsp*I was inactivated at 65°C for 20 min. The reactions were mixed with formamide loading buffer (95% formamide, 20 mmol L^−1^ EDTA pH 7.5) and loaded on polyacrylamide gels containing 7 mol L^−1^ urea. Samples were heated at 95°C for 10 min before loading. Afterwards, the gel was scanned and analyzed using the Odyssey^®^ Infrared Imaging System (Li‐Cor Biosciences, Bad Homburg, Germany). Percentage of repaired probe was calculated as described previously (Pokorny et al. 2008).

To study repair of (6–4)‐photoproducts by *U. maydis* Phr2, and repair of T<>T by Cry1, Cry2, and Phr1, an 18‐mer oligodT (Eurofins MWG Operon, Ebersberg, Germany) was used. To generate (6–4)‐photoproducts, 100 *μ*mol L^−1^ oligo(dT)_18_ in TE buffer (10 mmol L^−1^ Tris‐HCl, pH 7.5, 1 mmol L^−1^ EDTA) was irradiated (spectrofluorophotometer RF‐5301PC; Shimadzu) with *λ*_ex_ 260 nm (22 *μ*mol m^−2^ sec^−1^) for 135 min at 15°C. To generate the CPD lesion, oligo(dT)_18_ in TE buffer (10 mmol L^−1^Tris‐HCl pH 7.5, 1 mmol L^−1^ EDTA) was irradiated with UV transilluminator (TF‐20 mol L^−1^; Vilber Lourmat, Eberhardzell, Germany) as described previously (Pokorny et al. 2008). During irradiation, absorption changes at 265 nm and 325 nm were monitored spectroscopically (Gene Quant 1300, GE Healthcare). Decrease in 265 nm absorption originates from formation of both CPDs and (6–4)‐photoproducts, increase in 325 nm absorption from formation of (6–4)‐photoproducts (Kim and Sancar 1991; Yamamoto et al. 2013).

Repair assays of Phr2 contained 33 *μ*mol L^−1^ oligo(dT)_18_ with 4 CPDs (T<>T) and 1 T(6–4)T lesion per oligonucleotide in average and 0.7 *μ*mol L^−1^ prephotoreduced (fully reduced flavin state) *U. maydis* Phr2. Repair assays of Cry1 and Phr1 contained 5 *μ*mol L^−1^ oligo(dT)_18_ with 2.6 (Cry1) or 4.4 (Cry2 or Phr1) thymine dimers (T<>Ts) lesion per oligonucleotide in average, 50 nmol L^−1^ or 100 nmol L^−1^ prephotoreduced (fully reduced flavin state) purified protein. The final buffer conditions were 50 mmol L^−1^ Tris‐HCl pH 7.5, 50 mmol L^−1^ NaCl, 10% glycerol, 10 mmol L^−1^ DTT. Samples were placed in a Quartz Suprasil cell (Hellma GmbH & Co.KG) and irradiated with UV‐A (385 nm, 100 *μ*mol m^−2^ sec^−1^) by the spectrofluorophotometer (RF‐5301PC; Shimadzu) at 15°C. Spectra in the 240–450 nm range (Gene Quant 1300; GE Healthcare) were taken at different time points. Decrease in A_325_ was used to obtain the molar amount of repaired (6–4)‐photoproducts, increase at A_265_ for repair of T<>T (Kim and Sancar 1991). UV‐A‐treated reactions without enzyme served as control.

## Results

### *Ustilago maydis* contains ten photoreceptor candidate genes

**Figure 1:**
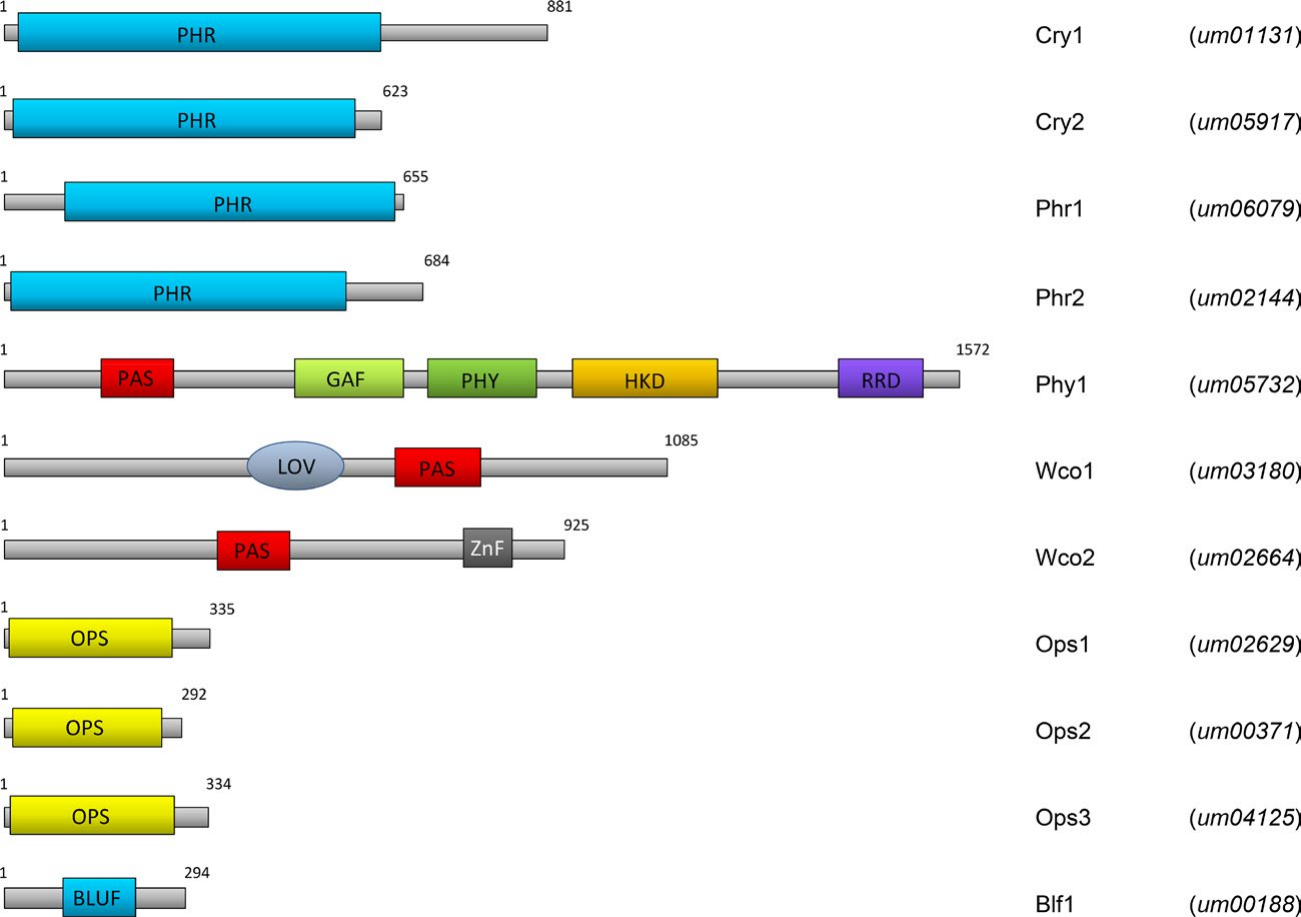
Domain structure of predicted light‐responsive proteins of *U. maydis*. Abbreviations are: PHR, Photolyase homology region; PAS, Per/Arnt/SIM domain; GAF, cGMP‐specific phosphodiesterase/Anabaena adenylate cyclase/*E. coli* FhlA domain; PHY, phytochrome‐specific domain; HKD, histidine kinase domain; RRD, response receiver domain; LOV, Light/Oxygen/Voltage domain; ZnF, zinc‐finger domain; OPS, opsin domain; BLUF, blue light sensing using FAD domain. The numbers indicate the length of the proteins in amino acids.

Searching the *U. maydis* genome database [http://pedant.helmholtz-muenchen.de/pedant3htmlview/pedant3view?Method=analysis&Db=p3_t237631_Ust_maydi_v2GB] for genes encoding putative photoreceptors revealed 10 genes fitting this criterion (Fig. 1). Four among these genes categorized to the CPF encoding two DASH‐type cryptochromes (*cry1*,* um01131; cry2, um05917*), a class I CPD‐photolyase (*phr1*,* um06079*), and a (6–4)‐photolyase (*phr2*,* um02144*). Among the other candidates, *um03180* (*wco1*) encodes a protein with the typical domain organization of White collar 1. Wco1 does not contain a zinc‐finger motif, which is typically present in other fungal clades, but not in Basidiomycetes (Idnurm and Heitman 2005). The PAS‐like domain in Wco1 contains a GKNCRFLQ motif, which is typical for LOV domains to bind the light‐sensitive flavin chromophore (Swartz and Bogomolni 2005). The second gene annotated as a *wc‐1* homolog (*um02052*) in the *Ustilago* database does not contain the typical hallmark of a LOV‐domain. Thus, we did not consider this gene as a photoreceptor candidate. A *wc‐2* homolog (*wco2*) is present in *U. maydis*, which is encoded by *um02664*. In addition, *U. maydis* encodes a protein with all characteristics of a fungal phytochrome (*phy1*,* um05732*). Moreover, there are three opsin‐like genes (*ops1*,* um02629; ops2, um00371*;* ops3*,* um04125*) in the *U. maydis* genome, which have been partially characterized (Estrada et al. 2009) and sometimes also annotated as small heat shock proteins (Ghosh 2014). Surprisingly, *U. maydis* also contains a BLUF‐domain protein encoded by *um00188* (blf1) as noticed before (Herrera‐Estrella and Horwitz 2007). BLUF‐domain proteins act as photoreceptors and are widely distributed in the bacterial kingdom (Losi and Gärtner 2012), but have not been described in other eukaryotes, except *U. maydis* and Euglenoids (Iseki et al. 2002). BLAST search revealed that BLUF‐proteins can also been found in species closely related to *U. maydis* such as *U. hordei*,* Sporisorium reilianum*,* Pseudozyma* sp., and *Melanopsichium pennsylvanicum*.

**Figure 2:**
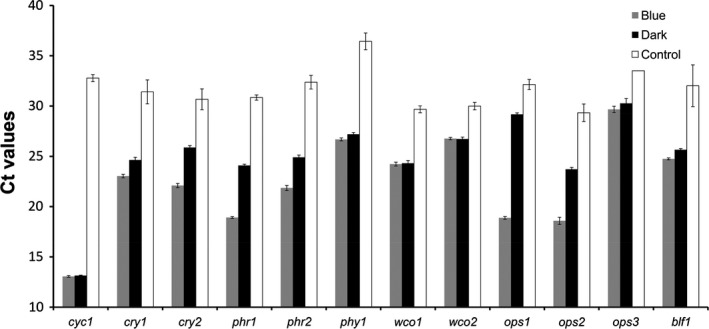
Photoreceptor and photolyase genes are expressed in *Ustilago maydis* axenic cultures. Shown are means and standard errors (*n* = 3) of Ct‐values of indicated transcripts from qRT‐PCR experiments. Samples were prepared from *U. maydis* wild‐type cells grown in liquid culture either in darkness (black bars) or treated with blue light (471 nm; fluence rate 30 *μ*mol m^−2^ sec^−1^) for 60 min (gray bars). Negative controls (white bars) included H_2_O instead of RNA. *cyc1* (cyclophilin, *um03726*) served as internal control. For abbreviations of genes see Fig. 1 and text.

In order to test the expression and response to blue light exposure of the 10 candidate genes, as well as of *wco2*, we performed qRT‐PCR on RNA isolated from *U. maydis* wild‐type cells grown in darkness or treated with blue light for 60 min. All candidate genes except *ops3* were expressed under these experimental conditions (Fig. 2), and all except *phy1*,* wco1*,* wco2*, and *blf1* were induced by blue light.

### *Ustilago maydis* is responsive to blue light

Responsiveness to blue light is well documented for several fungal species such as *N. crassa*,* P. blakesleeanus, A. nidulans*, and *F. fujikuroi* (Herrera‐Estrella and Horwitz 2007; Idnurm et al. 2010; Rodriguez‐Romero et al. 2010). In order to investigate blue light‐mediated responses in *U. maydis*, transcriptome analysis was performed using Affymetrix‐based microarrays (Kämper et al. 2006). *U. maydis* wild‐type cells were grown in continuous darkness or treated with blue light at a fluence rate of 30 *μ*mol m^−2^ sec^−1^ for 60 min. Extracted RNA samples of three biological replicates were labeled and hybridized to the microarray chips. Normalization and statistical analysis of the expression data identified 61 transcripts being differentially regulated between dark and light‐treated samples (≥twofold difference in expression; *P* ≤ 0.05) (Table 1). While only one gene was downregulated, 60 genes were transcriptionally induced, corresponding to about 1% of the *5824 U. maydis* genes represented on the microarray. Among the upregulated genes were three of the four CPF members namely *phr1* (*um06079*), *cry2* (*um05917*), and *phr2* (*um02144*) with fold‐induction values of 20.8, 9.5, and 5.2, respectively (Table 1).

**Table 1:** Blue light‐controlled genes of *Ustilago maydis* wild‐type and *Δwco1* mutant

Gene	Annotation	Blue light induction wild‐type	Blue light induction Δ*wco1*
*um10690*	Hypothetical protein	121.84	4.48
*um02629*	Related to YRO2 ‐ putative plasma membrane protein, transcriptionally regulated by Haa1p	79.87	–
*um10676*	Conserved hypothetical protein	59.23	–
*um02723.2*	Probable mfs‐multidrug‐resistance transporter	45.01	7.70
*um00286*	Hypothetical protein	34.91	5.62
*um10657*	Conserved hypothetical protein	32.43	8.37
*um05328*	Conserved hypothetical protein	31.79	10.88
*um11403*	Conserved hypothetical protein	27.64	–
*um10208*	Conserved hypothetical protein	23.63	3.64
*um06079*	Related to deoxyribodipyrimidine photolyase PHR	20.78	6.91
*um10865*	Conserved hypothetical protein	19.32	3.12
*um00371*	Related to Opsin‐1	17.24	–
*um01815*	Related to carbonyl reductase	16.93	–
*um00719*	Hypothetical protein	13.70	–
*um11249*	Related to cyclopropane‐fatty‐acyl‐phospholipid synthase	13.16	–
*um03485*	Conserved hypothetical protein	12.31	–
*um04575*	Conserved hypothetical protein	11.73	–
*um10868*	Conserved hypothetical protein	11.18	–
*um05917*	Related to deoxyribodipyrimidine photolyase	9.52	–
*um04712*	Related to N‐methyltransferase	9.31	–
*um03016*	Conserved hypothetical protein	7.14	–
*um00205*	Related to HSP12‐heat shock protein	6.90	–
*um06063*	Related to GAD1 ‐ glutamate decarboxylase	6.64	–
*um00573*	Conserved hypothetical protein	6.25	–
*um03556*	Conserved hypothetical protein	6.06	–
*um00749*	Related to lipase	5.97	–
*um11229*	Conserved hypothetical protein	5.87	–
*um06119*	Conserved hypothetical protein	5.61	–
*um03994*	Probable PDC1 – pyruvate decarboxylase, isozyme 1	5.56	–
*um04742*	Related to stomatin	5.52	–
*um03779*	Related to galactinol synthase	5.39	–
*um02070*	Conserved hypothetical protein	5.32	–
*um02144*	Related to deoxyribodipyrimidine photolyase	5.17	–
*um04005*	Conserved hypothetical protein	4.82	–
*um10062*	Related to monooxygenase	4.80	–
*um05961*	Probable alpha‐methylacyl‐coa racemase	4.42	–
*um04724*	Conserved hypothetical protein	3.92	–
*um03177*	Related to peroxisomal membrane protein 20	3.83	–
*um10002*	Related to NADH‐dependent flavin oxidoreductase	3.80	–
*um04922*	Related to 2,5‐diketo‐D‐gluconic acid reductase	3.63	–
*um10540*	Related to blue‐light‐inducible Bli‐3 protein	3.61	–
*um02161*	Conserved hypothetical protein	3.58	–
*um03506*	Conserved hypothetical protein	3.56	–
*um02876*	Conserved hypothetical protein	3.43	–
*um11978*	Conserved hypothetical protein	3.42	–
*um02721*	Conserved hypothetical protein	3.38	–
*um05222*	Putative protein	3.36	–
*um02888*	Related to ADH6 ‐ NADPH‐dependent alcohol dehydrogenase	3.35	–
*um01351*	Putative protein	3.29	–
*um10780*	Conserved hypothetical protein	3.20	–
*um10692*	Putative protein	3.16	–
*um01728.2*	Conserved hypothetical protein	2.90	–
*um04749*	Conserved hypothetical protein	2.89	–
*um04947*	Putative protein	2.78	–
*um10392*	Conserved hypothetical protein	2.75	–
*um01185*	Conserved hypothetical protein	2.73	–
*um06428*	Related to Thiamine‐repressible acid phosphatase precursor	2.68	–
*um03073*	Related to GTT1 – glutathione–S‐transferase	2.64	–
*um01724*	Conserved hypothetical protein	2.58	–
*um10207*	Related to AMD2 ‐ acetamidase	2.34	–
*um04910*	Conserved hypothetical protein	−3.20	–

Members of the cryptochrome/photolyase family (CPF) are highlighted.

**Figure 3:**
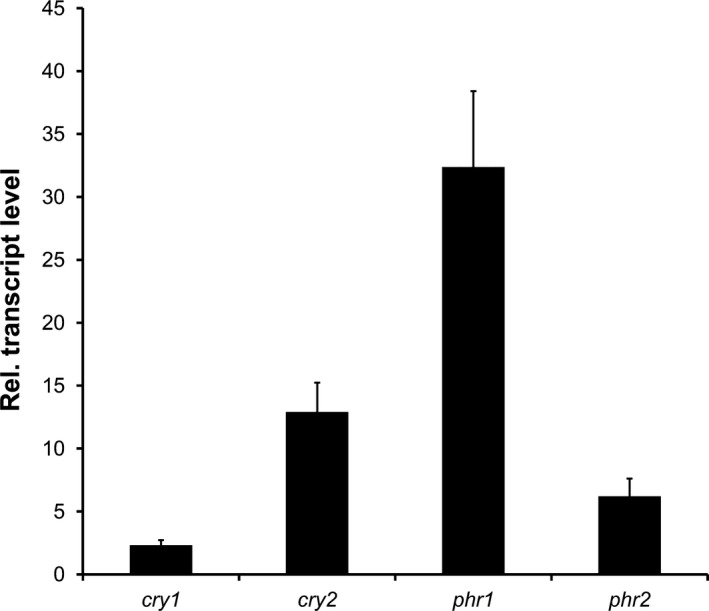
Expressions of cryptochrome/photolyase genes are induced by blue light. Transcript levels of the *U. maydis* CPF members were quantified by qRT‐PCR in samples from wild‐type cells grown in darkness or treated for 1 h with blue light (471 nm, 30 *μ*mol m^−2^ sec^−1^). Given are the values of light samples normalized against the dark control. Data represent mean and standard errors from three biological replicates.

Blue light induction of the CPF members was confirmed by qRT‐PCR (Fig. 3). Although the values of induction differed between the two techniques, transcript levels of *phr1*,* cry2*, and *phr2* were significantly higher in blue light‐exposed cells than in cells grown in darkness. Moreover, qRT‐PCR data showed a weak (2.4‐fold), but significant induction of *cry1* (Fig. 3) that was not seen in the microarray analyses. These data demonstrate that *U. maydis* is responsive to blue light and that all members of the CPF are upregulated by blue light.

### White collar 1 acts as a blue light photoreceptor in *U. maydis*

**Figure 4:**
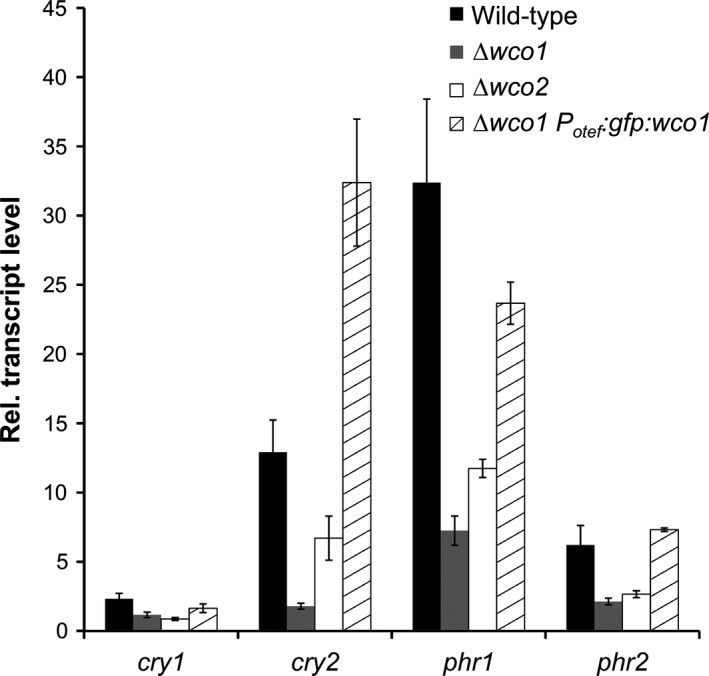
Blue light induction of cryptochrome/photolyase genes is controlled by Wco1. Shown are transcript levels quantified by qRT‐PCR of cells irradiated with blue light (471 nm, 30 *μ*mol m^−2^ sec^−1^) normalized against dark controls of the respective genotype. Analyzed genotypes were wild‐type (black bars), *Δwco1* (gray bars), *Δwco2* (white bars), and *Δwco1* complemented with GFP‐Wco1 (hatched bars). Given are means and standard errors (*n* = 3).

In *N. crassa*, WC‐1 is forming together with WC‐2, a photoresponsive transcription factor complex (WCC) which regulates gene expression in a light‐dependent fashion (Chen et al. 2010). The role of the orthologous genes *wco1* and *wco2* in light responses has not been investigated in *U. maydis* so far. We analyzed the transcriptome of *U. maydis* wild‐type versus *Δwco1* deletion mutant cells in dark‐grown and under blue light‐treated conditions. Only eight genes showed blue light‐induced differential expression in the *Δwco1* mutant at a significant threshold level above two (Table 1). All of these eight genes were induced and belong to the strongly induced genes in the wild type. However, the fold induction of these genes was much smaller in *Δwco1* than in the wild‐type. To reaffirm the array data, we quantified the transcript levels of the CPF members by qRT‐PCR. As shown in Figure 4, the blue light‐driven transcript induction in *Δwco1* was either completely abolished (*cry1*,* cry2*,* phr2*) or strongly reduced (*phr1*).

To exclude the possibility that deletion of *wco1* had caused some side effects, we transformed this strain with a P_*otef*_:*gfp*:*wco1* construct and tested for complementation of the *Δwco1* phenotype. The ectopic expression of Wco1 could revert the blue light‐induced transcripts similar to wild‐type levels (Fig. 4). Together, these data clearly show that Wco1 is the main blue light photoreceptor in *U. maydis* at least under the applied experimental conditions. Since *Δwco1* still shows induction of a few genes, we assume that other and so far undefined blue light photoreceptors exist in *U. maydis*.

To figure out whether Wco1 requires functional Wco2 also in *U. maydis*,* wco2* knockout mutants were constructed and analyzed for blue light induction of CPF members. Indeed, the expression levels of these genes were reduced in *Δwco2* cells to a very similar extent as in *Δwco1* (Fig. 4). This supports the notion that in *U. maydis,* Wco1 operates together with Wco2 as a blue light‐dependent transcription factor.

### White collar 1 and White collar 2 localize to the nucleus and function as a complex

**Figure 5:**
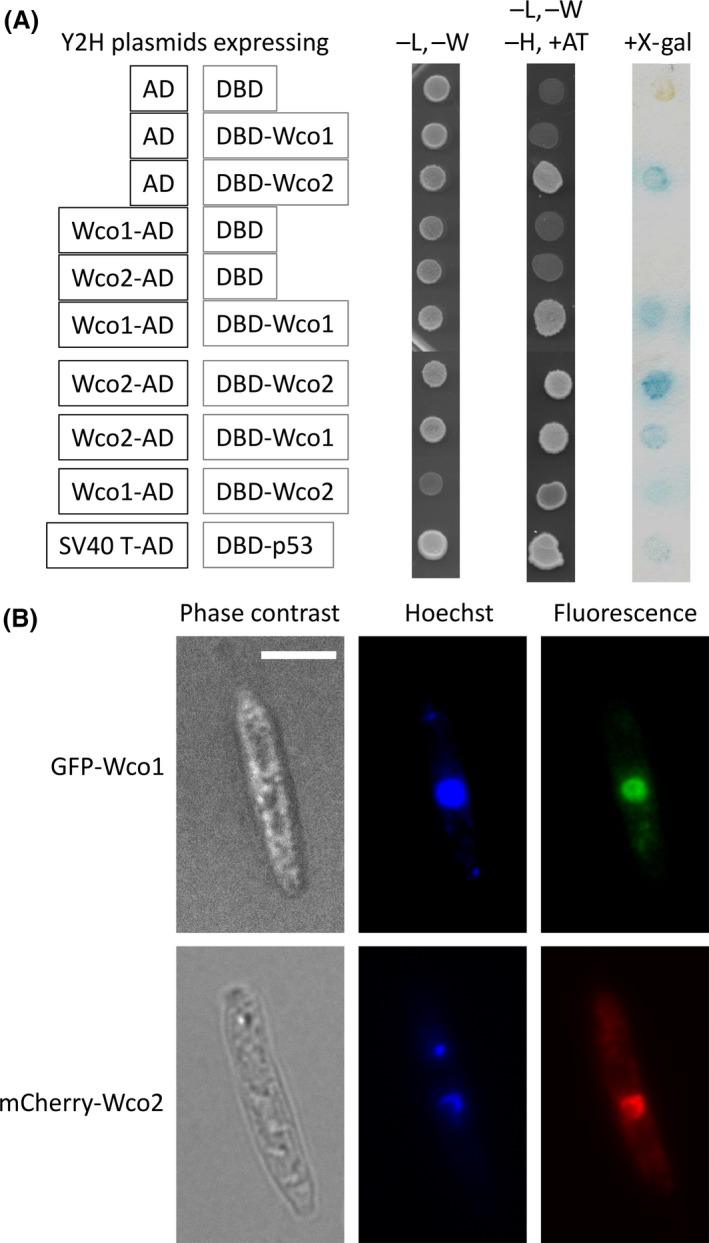
*Ustilago maydis* Wco1 and Wco2 are nuclear proteins and interact with each other. (A) Wco1 or Wco2 proteins were expressed as fusions of GAL4 activation domain (AD), GAL4 DNA‐binding domain (BD) and their interactions were tested by yeast‐ two‐hybrid assay. Yeast transformants were spotted on minimal medium lacking indicated amino acids, along with positive control plasmids provided by the supplier. The positives for protein–protein interactions were determined by growth on selective medium SD,‐L,‐W,‐H with 3‐aminotriazole (3‐AT) and activation of the ß‐galactosidase was tested with the X‐gal substrate by a filter lift assay. (B) Wco1 and Wco2 were expressed in *U. maydis* wild type as a fusion to the C‐terminus of a fluorescent protein from a constitutive *otef* promoter. Fluorescence microscopic images showing exclusive or enriched nuclear localization of GFP‐Wco1 and mCherry‐Wco2, respectively, and the phase contrast images. Nuclei were visualized by Hoechst 33,342 dye staining. Bar, 5 *μ*m for all pictures.

We used the yeast‐two‐hybrid (Y2H) system to test for Wco1/Wco2 complex formation. *wco1* and *wco2* were fused to the Gal4‐activation domain (AD‐X) or the DNA‐binding domain (BD‐X) in all possible combinations (Fig. 5A). Whereas coexpression of BD‐Wco1 with the empty AD‐vector did not show activation of the *β*‐galactosidase reporter, the BD‐Wco2/empty AD‐vector combination did so. This indicates that Wco2 has transactivation activity, which is typical for a transcription factor. When fused to the Gal4‐AD, Wco2 did not show activation of the reporter and selection marker. Thus, the AD‐Wco2 construct was used to study interaction between Wco1 and Wco2. Indeed, we observed activation of the *His* and *lacZ* reporter genes with the AD‐Wco2/BD‐Wco1 combination (Fig. 5A) clearly demonstrating direct interaction between Wco1 and Wco2. Moreover, activation of the reporters in yeast cells expressing the AD‐Wco1/BD‐Wco1 combination indicates homooligomerization of Wco1. Whether Wco2 also can form oligomers could not be tested by our Y2H because of the above mentioned transactivation activity of the BD‐Wco2 fusion.

Light‐induced gene activation by WCC in *N. crassa* is mediated by direct binding of WCC to light‐responsive elements in the promoter regions of the induced genes (Crosthwaite et al. 1997; Chen et al. 2009). Therefore, we tested whether Wco1 and Wco2 also localized to the nucleus in *U. maydis*. Constructs of Wco1 fused with GFP (GFP‐Wco1) and of Wco2 fused with mCherry (mCherry‐Wco2) driven by the constitutive *otef* promoter (Hartmann et al. 1999) were stably expressed in *U. maydis* wild‐type cells. Fluorescence microscopic studies showed nuclear localization of GFP‐Wco1 (Fig. 5B). Signals of mCherry‐Wco2 were detected both in the nucleus and in the cytosol (Fig. 5B). Taken together, these studies demonstrate that *U. maydis* Wco1 and Wco2 form a WCC acting as a nuclear blue light photoreceptor similar as in other fungal species (Chen et al. 2010).

### CPD‐ and (6–4)‐photolyases contribute to UV‐B tolerance of *U. maydis*

*U. maydis* is known to be highly resistant to UV‐B and ionizing radiation. Its efficient recombination repair also is well documented (Holloman et al. 2007, 2008). However, it was not known whether photolyase‐mediated photoreactivation contributes to the UV‐tolerance of *U. maydis*. We use the term photoreactivation in its original definition (Kelner 1949) as an increase in the number of surviving cells as a consequence of UV‐A or visible light given after UV‐B exposure. The fact that *U. maydis* possess four members of the CPF prompted us to analyze whether *U. maydis* is able to photoreactivate, and if so, which role each of the CPF members plays in this process.

**Figure 6:**
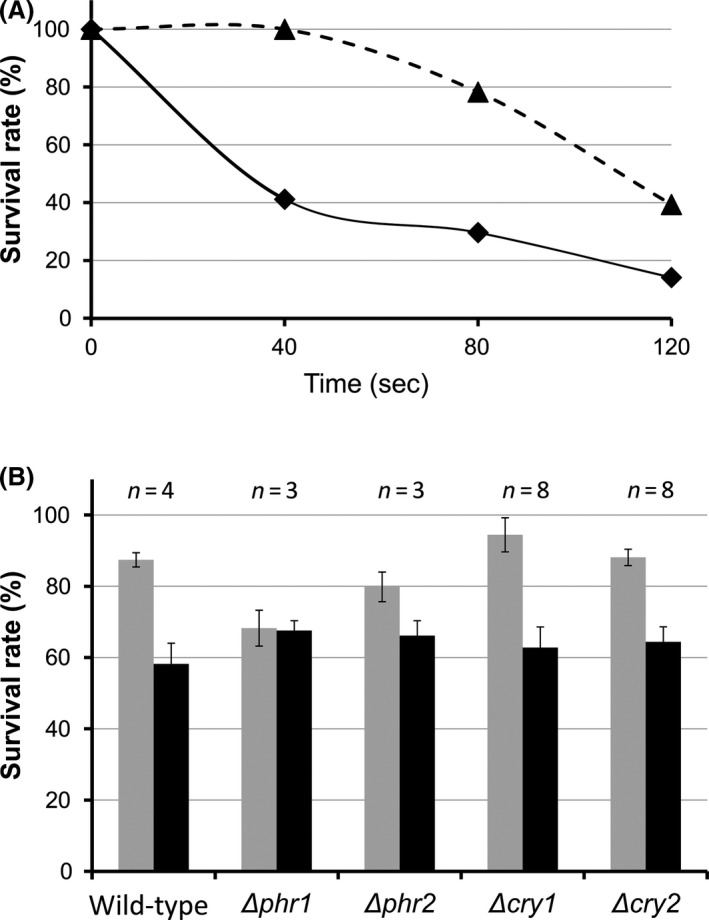
Photoreactivation of *Ustilago maydis* is mediated by Phr1 and Phr2. (A) Survival rate of *U. maydis* wild type treated with increasing doses of UV‐B (for spectrum see Fig. S1). Cells were exposed to UV‐B with irradiation times indicated and transferred directly to darkness (solid line) or allowed for phototoreactivation (broken line). (B) Survival rate of wild‐type and CPF member mutants treated with UV‐B for 40 sec and given no (black bars) or 1 h photoreactivating light (white bars). Data of biological replicates as indicated (*n* = 3–8). Wild‐type and CPF mutants Δ*phr1*, Δ*phr2,* Δ*cry1*,* and* Δ*cry2*.

To test for photoreactivation, wild‐type cells were spread on PD‐plates, irradiated with UV‐B and subsequently transferred to darkness or allowed for photoreactivation under white light for 1 h followed by 48 h incubation in the dark. Colony counting showed a decrease in survival rate at higher UV‐B doses. By contrast, survival rates were strongly increased when cells were incubated with white light after UV‐B treatment (Fig. 6A). This result unambiguously shows photoreactivation by *U. maydis*. To find out which one of the four CPF members is required for photoreactivation, deletion mutants for each gene were generated and their survival rates analyzed after UV‐B irradiation followed by dark incubation or white light treatment (Fig. 6B). Wild‐type cells, Δ*cry1*, and Δ*cry2* showed undistinguishable photoreactivation behavior, suggesting that none of the DASH‐type cryptochromes contributes to photoreactivation of *U. maydis*. In contrast, disruption of *phr1* completely abolished photoreactivation (Fig. 6B). This demonstrates that the encoded protein plays an essential role in photoreactivation of *U. maydis*. The mutant of *phr2* had an intermediate phenotype between wild‐type and the CPD‐photolyase mutant (Fig. 6B), indicating that repair of CPD‐lesions by CPD‐photolyase in *U. maydis* has a priority over the repair of (6–4)‐photoproducts by the (6–4)‐photolyase.

### *U. maydis* CPF members bind flavin, are photoactive and repair UV‐lesions in vitro

Data shown in Fig. 6 strongly suggest that at least the predicted CPD‐ and (6–4)‐photolyase have an in vivo function as DNA repair enzymes in *U. maydis*. One essential prerequisite for a photolyase is binding of the FAD cofactor. If this cofactor is not in the fully reduced anionic state (FADH^−^), which is the only known catalytic state upon photoexcitation, its reduction or photoreduction becomes likewise essential (Sancar 2003). To test for the presence of cofactors and observe FAD‐photoreduction, the four CPF members of *U. maydis* were expressed and purified as His‐ and Strep‐tag fusions from *E. coli*. All proteins were purified close to homogeneity in soluble form and had the expected molecular masses (Fig. S2A). The identity of the proteins was further confirmed by immunoblotting using His‐tag antibodies and mass spectrometry (data not shown). UV‐Vis absorption spectroscopy showed that the four CPF members bind chromophores as seen by the absorbance in UV‐A and the visible range of the spectrum (Fig. S2B–E). Absorbance in the range between 440 nm and 500 nm with fine structures and peaks at around 445 nm and shoulders around 470 nm is typical for the fully oxidized state of FAD (Zirak et al. 2009). A strong and higher absorbance than in the 450 nm range was observed for Cry2, Phr1, and Phr2 at around 380 nm (Fig. S2C–E). Usually, this is typical for protein‐bound MTHF, a common antenna of photolyases (Sancar 2003). In case of Cry1, we observed in the 380 nm range a peak of similar height as in the 450 nm range (Fig. S2B). This indicates that *U. maydis* Cry1 does not bind MTHF in contrast to Cry2 and Phr1. The absorption spectrum of Phr2 in the 330 nm–400 nm region (Fig. S2E) does not fit well with that of MTHF‐binding CPF members and suggests the presence of an unknown cofactor that needs to be identified in the future. Illumination with blue light resulted in absorption changes in all four CPF members (Fig. S3). These changes are consistent with lifetimes of fully oxidized FAD in the range of minutes and with the formation of fully reduced flavin (FADH^−^/FADH_2_). Reoxidation to FAD_ox_ was much slower and occurred in the range of hours (data not shown). Together, these data proved that all CPF members of *U. maydis* bind FAD. Methenyltetrahydrofolate (MTHF) is the second cofactor of Cry2 and Phr1. Moreover, photoreduction studies showed that all CPF members reduce FAD_ox_ to fully reduced FADH^−^/FADH_2_ and thus are photoactive proteins.

**Figure 7:**
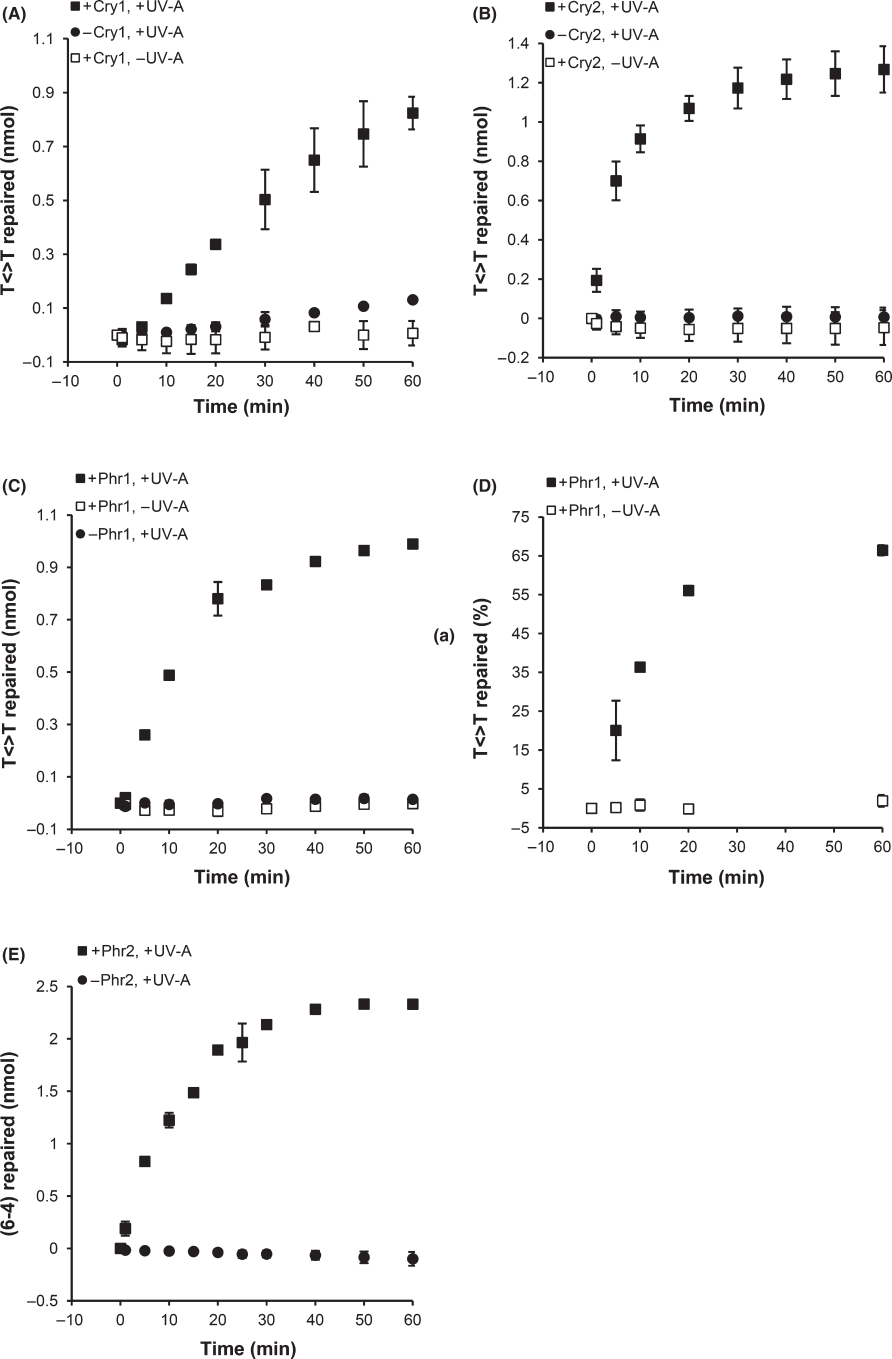
CPF members of *U. maydis* repair UV‐lesions in vitro. (A) Kinetics of repair of T<>T in an oligo(dT)_18_ in the absence or presence of Cry1. The curves show the calculated molar amounts of repaired T<>T in the different assays: samples containing 50 nmol L^−1^ Cry1 and treated with photoreactivating UV‐A (black squares); samples containing 50 nmol L^−1^ Cry1 incubated in darkness (white squares); samples containing no Cry1 and treated with photoreactivating UV‐A (circles). Given are means and standard errors of two independent experiments. (B) Kinetics of repair of T<>T in an oligo(dT)_18_ in the absence or presence of 100 nmol L^−1^ Cry2. The curves show the calculated molar amounts of repaired T<>T in the different assays. Symbols in curves are as in A except that Cry2 was used. Given are means and standard errors of two independent experiments. (C) Repair of T<>T in ssDNA by 100 nmol L^−1^ Phr1. Symbols in curves are as in A. (D). Repair of T<>T in dsDNA by Phr1. The curves show the calculated percentage amounts of repaired T<>T in the different assays: samples containing 40 nmol L^−1^ PHR1 and treated with photoreactivating UV‐A (black squares); samples containing 40 nmol L^−1^ Phr1 incubated in darkness (white squares). Given are means and standard errors of two independent experiments. (E) Repair of (6–4)‐photoproducts by Phr2. Curves show the repair kinetics in the absence (circles) or presence of 0.7 *μ*mol L^−1^ Phr2 (squares). Samples were treated with UV‐A to allow repair. Given are means and standard errors of two independent experiments.

We went ahead to characterize the *U. maydis* CPF members for their ability to repair pyrimidine dimers. DASH‐type cryptochromes are known to repair CPDs in single‐stranded DNA (ssDNA), but not in double‐stranded DNA (dsDNA) (Selby and Sancar 2006). We performed in vitro assays to observe repair of CPDs in a single‐stranded (ss) oligo(dT)_18_. The assays were performed in the presence and absence of photoreactivating light. Both, Cry1 (Fig. 7A) and Cry2 (Fig. 7B) showed repair activity of CPDs exclusively in the light‐exposed samples. Thus, *U. maydis* Cry1 and Cry2 behave similar to other DASH‐type cryptochromes with regard to repair of CPDs in ssDNA. Further, we tested the ability of Phr1 to repair CPDs in ssDNA and dsDNA probes. As expected from the severe phenotype of the *U. maydis* Δ*phr1* mutant (Fig. 6B), we observed light‐driven repair of CPDs in ssDNA (Fig. 7C) as well as in dsDNA (Fig. 7D), thus assigning Phr1 a canonical class I CPD‐photolyase. Likewise, the ability of the predicted (6–4)‐photolyase to repair (6–4)‐photoproducts was analyzed by an in vitro assay using (6–4)‐lesion‐containing substrate. We observed light‐driven repair of (6–4)‐photoproducts (Fig. 7E) confirming that *phr2* encodes a functional (6–4)‐photolyase.

### Wco1 contributes to UV‐tolerance of *U. maydis*

**Figure 8:**
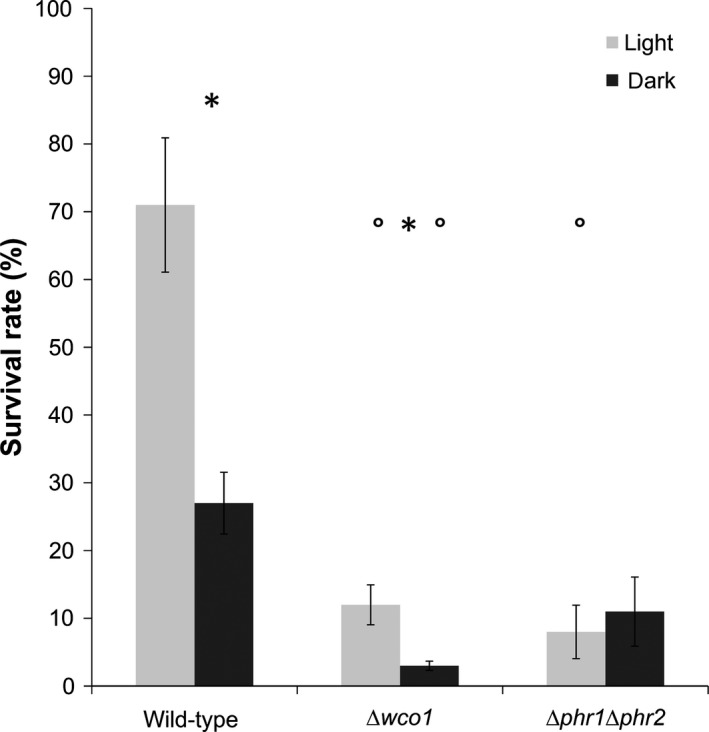
Wco1 contributes to UV‐resistance of *Ustilago maydis*. Survival rate of *U. maydis* wild‐type, Δ*wco1*,* and* Δ*phr1/*Δ*phr2* mutants upon UV‐B treatment (330 J m^−2^) followed by absence (black bars) or presence (gray bars) of photoreactivating light. Data represented are means with standard errors from three biological replicates. *Indicates significant difference (*P* ≤ 0.05) between light and dark samples of the same genotype; °Indicates significant difference (*P* ≤ 0.05) between wild‐type and the mutant of the same light program.

The light‐induced upregulation of CPF members seen in wild‐type is significantly reduced or even abolished in the *Δwco1* mutant (Table 1, Fig. 4). This provoked us to address the question whether the UV‐tolerance of *U. maydis* is reduced in the *Δwco1* mutant. Compared to wild type, the survival rate of *Δwco1* under UV‐B is strongly reduced. However, *Δwco1* shows an increased survival rate in light compared to darkness, indicating that its total capacity to survive the UV‐B treatment is reduced, but not completely abrogated (Fig. 8). As expected, the Δ*phr1*Δ*phr2* double mutant showed a similar survival rate in light and darkness confirming the in vitro data and supporting the concept that cry‐DASHs play no obvious role in photoreactivation of *U. maydis*. We conclude that the reduced expression of *phr1* and *phr2* in the *Δwco1* mutant (Fig. 4) is one of the important factors responsible for its increased sensitivity against UV‐radiation.

## Discussion

In contrast to several other fungal species, there are only few publications addressing the question of how *U. maydis* responds to light (e.g., Estrada et al. 2009). Moreover, none of the known photoreceptor systems has been analyzed in this fungus. Therefore, this study aims to set the basis for a future photobiology of *U. maydis*. *U. maydis* has the full set of photoreceptors known from other fungi, and, in addition, a gene encoding a BLUF‐domain protein (Blf1) which is very uncommon to eukaryotes (Fig. 1). This is in contrast to the basidiomycete *C. neoformans,* which encodes only three photoreceptor candidates (*BWC1*,* PHY1*, and *OPS1*) (Idnurm and Heitman 2005). Importantly, we could verify the expression of nine of the ten putative photoreceptor candidates in *U. maydis* wild types (Fig. 2). The only photoreceptor candidate whose expression was not detected (*um04125*), previously described as *ops3* (Estrada et al. 2009), is annotated as a heat shock protein. While this gene is not expressed in axenic cultures, it was found to be strongly induced during host infection (Ghosh 2014).

Most of the photoreceptor candidate genes in *U. maydis* are predicted to bind flavin (Wco1, CPF members, Blf1) and thus should absorb in the UV‐A/blue‐light region of the spectrum. Therefore, we decided to test specifically the global transcriptional response of *U. maydis* to blue light, and identified 60 genes induced and one gene repressed by blue light based on a twofold threshold level (Table 1). The number of blue light‐controlled genes in *U. maydis* is much higher than in *C. neoformans* using the same approach and stringent cutoff, which revealed only one gene encoding ferrochelatase to be controlled by white light (Idnurm and Heitman 2010). In *N. crassa*, 5.6% of the total detectable transcripts are under control of white light including those of early and late responding genes (Chen et al. 2009). We assume that the real number of light‐responsive genes in *U. maydis* is higher than described here for the following reasons: 1. We used a very stringent cutoff for the analysis of array data; 2. We checked the specific role of blue light thus excluding activation of rhodopsins and phytochrome, which operate in the green and red spectral range, respectively (Blumenstein et al. 2005; Brandt et al. 2008; Purschwitz et al. 2008; Garzia‐Martinez et al. 2015); 3. We sampled only at one time point (60 min) after light on and thus could have missed transcripts expressed late or very transient as has been described for *N. crassa* (Chen et al. 2009). Studies are in progress to identify genes, which are controlled by other wavebands and/or are expressed at other time points.

The transcriptional response revealed that only eight of the 61 blue light‐controlled genes in wild‐type are differentially expressed in *Δwco1* mutants (Table 1). Moreover, these eight genes showed strongly decreased induction ratios (e.g., *um06079*: 24‐fold in wild‐type and threefold in *Δwco1*). These data unambiguously show that Wco1 has the most prominent role in blue light perception of *U. maydis* as in other fungi such as *N. crassa* (Chen et al. 2009), *C. neoformans* (Idnurm and Heitman 2005, 2010), *P. blakesleeanus*,* and M. circinelloides* (Corrochano and Garre 2010). However, the residual blue light induction of few genes in *Δwco1* suggests presence of other photoreceptors which also mediate blue light regulation. We consider the second gene annotated as a *wc‐1* homolog (*um02052*) in the *Ustilago* database as the most unlikely candidate since the encoded protein does not contain the conserved GKNCRFLQ motif in its PAS domain, which is required for binding and covalent linkage of the light‐sensitive flavin chromophore (Swartz and Bogomolni 2005), but we cannot completely rule out its role as photoreceptor. Other putative blue light photoreceptors in *U. maydis* are the four members of the CPF and the BLUF‐domain protein. Moreover, *U. maydis* encodes a protein (um04464.1) with sequence homology to Dst2 from *C. cinerea*. Dst2 has a split FAD‐binding‐4 domain and its mutant is severely affected in blue light perception (Kuratani et al. 2010). However, FAD‐binding to Dst2 is not documented and we have therefore not included um04464.1 in our list of putative light‐responsive proteins. A photoreceptor function was shown for cry‐DASH in other fungi including *N. crassa*,* S. sclerotiorum*, and *F. fujikuroi* (Fröhlich et al. 2010; Olmedo et al. 2010; Nsa et al. 2015; Veluchamy et al. 2008; Castrillo et al. 2013) and for the CPD‐photolyase in *A. nidulans* (Bayram et al. 2008). We cannot exclude that CPF members in *U. maydis* also are involved in light regulation, despite their DNA repair activities demonstrated here. The same applies for the BLUF‐domain protein. This requires further studies to analyze light responses in mutants of the respective genes. However, the weak response to blue light of the *Δwco1* mutant clearly shows that such a suspected role is rather minor at least under the experimental conditions.

WC‐1 in *N. crassa* is known to form heterodimers with WC‐2 resulting in the white collar complex (WCC), and to act as light‐regulated transcription factor which binds to light‐responsive elements in the promoter regions at least of early induced genes (Ballario et al. 1996; Crosthwaite et al. 1997; Chen et al. 2009). The homologous proteins BWC1 and BWC2 from the basidiomycete *C. neoformans* dimerize as well and have a light‐dependent role on UV‐resistance and filamentation, but a light‐independent function on virulence (Idnurm and Heitman 2005). Interestingly, the WC‐1 homologs of basidiomycetes lack a zinc‐finger motif in contrast to Ascomycetes (Idnurm and Heitman 2005) and Mucormycotina such as *Phycomyces* (Corrochano and Garre 2010), but the zinc‐finger motif is conserved in WC‐2 homologs of all fungal clades including basidiomycetes (Fig. 1). Thus, a role of WCC in DNA‐binding and direct transcriptional control of target genes is very likely also for *U. maydis*. Indeed, we could show by fusion with fluorescent proteins that Wco1 and Wco2 localize to the nucleus and form a complex based on Y2H assays (Fig. 5). Furthermore, our Y2H assays suggest that Wco1 also can interact with itself. Whether this has biological relevance remains to be clarified. However, we assume that Wco1 requires Wco2 also in *U. maydis* because *Δwco2* has a similarly reduced blue light response as *Δwco1* (Fig. 4).

We screened the upstream intergenic region of the blue light‐induced and Wco1‐regulated CPF genes for the presence of any potential consensus signature elements using the analysis tool “The MEME suite” (Bailey and Elkan 1994). The analyzed data revealed the consensus motif GATVC….CGATV (where V can be any nucleotide except T). This motif is present in all upregulated genes except *um03994*,* um05961*, and *um02161*. The spacing between the two motifs varies significantly. Thus, the conserved element in blue light‐induced *U. maydis* genes resembles the light‐responsive element of *N. crassa* (GATNC….CGATN) except that N can be any nucleotide (He and Liu 2005).

Whether the three genes mentioned above are not under direct control of the WCC remains to be investigated in the future as well as binding of WCC to this element.

Classification of genes upregulated by blue light into functional categories revealed that genes involved in cell rescue, defense, and virulence as well as genes classified to be involved in interaction with the environment are overrepresented whereas genes of most other categories are underrepresented (data not shown). This suggests that light serves as a signal for *U. maydis* to adapt to adverse environmental conditions including exposure to UV‐radiation as described for other fungi (Rodriguez‐Romero et al. 2010; Fuller et al. 2015). Indeed, we found three of the four CPF members in *U. maydis* including *phr1* and *phr2* among the light‐induced genes (Table 1). We confirmed these data by qRT‐PCR (Fig. 3) and found an additional CPF member (*cry1*,* um01131*) to be 2.4‐fold upregulated by blue light treatment. This raised the question whether *U. maydis* encodes functional photolyases and in consequence is able to photoreactivate.

Studies with radiation‐sensitive mutants of *U. maydis* in the early 1970s used the activity of nitrate reductase as a marker for monitoring DNA repair. They observed that UV‐B‐repressed expression of nitrate reductase is enhanced by UV‐A after the UV‐B exposure (Resnick and Holliday 1971). However, the genes involved were unknown. From our studies, we could confirm their observation that *U. maydis* responds to photoreactivating light, which had a significant positive effect on survival after UV‐B exposure (Fig. 6A). Here, we could demonstrate that Δ*phr1* and Δ*phr2* show no and reduced photoreactivation, respectively, whereas the two *cry‐DASH* (Δ*cry1* and Δ*cry2*) mutants are identical to wild‐type (Fig. 6B). This supports the notion that Phr1 and Phr2 are functional photolyases, and is first clear evidence that these enzymes mediate photoreactivation of *U. maydis*. The fact that we did not observe any difference in photoreactivation between Δ*phr1* single and Δ*phr1*Δ*phr2* double mutants suggests that repair of (6–4)‐photoproducts by Phr2 is less important as long as CPDs remain unrepaired. Compared to wild‐type, the survival rate of Δ*wco1* mutants was lower when treated only with UV‐B (Fig. 8). At first glance, this is surprising since this result suggests a role of Wco1 in darkness. We assume, however, that the residual UV‐A and visible light in the UV‐B source (see spectrum Fig. S1) could allow activation of additional repair systems. However, transcript profiling gave no support for such an assumption.

The photolyase function of Phr1 and Phr2 was further supported by our biochemical characterization of the recombinant CPF members. The absorption spectra of dark‐incubated samples of all CPFs (Fig. S2) showed peaks or shoulders at around 450 nm and 475 nm indicative for the presence of fully oxidized flavin and, more or less pronounced, absorption in the range between 500 nm and 650 nm indicating the presence of the neutral flavin radical as seen in other photolyases and cryptochromes (Chaves et al. 2011). This is clear evidence that the four *U. maydis* CPF members bind the flavin cofactor essential for catalysis in photolyases and light signaling of cryptochromes (Sancar 2003). Moreover, the strong peak at around 380 nm seen for all *U. maydis* CPFs except Cry1 strongly suggests the presence of MTHF known to function as antenna chromophore (Chaves et al. 2011) or an unknown cofactor in case of Phr2. We modeled the structure of *U. maydis* Cry1 based on the structure of cry‐DASH (cry3) from *Arabidopsis thaliana* (Klar et al. 2007) and found residues such as Glu149 of cry3, which is essential for MTHF binding (Zirak et al. 2009) conserved in Cry1. However, additional loops in this region could interfere with MTHF‐binding. The UV‐Vis spectrum of Phr2 differs from spectra of known MTHF‐binding CPFs. In contrast to the MTHF peak usually found at around 380 nm, the UV‐A peak of Phr2 is at 369 nm. Furthermore, peaks typical for FAD_ox_ at 445 nm and 475 nm are superimposed on the above UV‐A peak. This is reminiscent, but not identical to the case of prokaryotic CPF proteins with iron–sulfur cluster (Oberpichler et al. 2011) where the absorption spectrum of FAD_ox_ is superimposed on the spectrum of a cofactor later identified as 6,7‐dimethyl‐8‐ribityl‐lumazine (Geisselbrecht et al. 2012; Zhang et al. 2013). Given that a fluorescence analysis of *U. maydis* Phr2 did not support the presence of MTHF (data not shown), we conclude that this protein contains besides FAD an additional cofactor whose identification must await further studies.

Most importantly, all four *U. maydis* CPFs respond to blue light (photoreduction of the flavin cofactor) concomitant with formation of fully reduced FAD (Fig. S3) which is required for photolyase to be catalytically competent (Sancar 2003). Thus, the spectroscopic behavior of the *U. maydis* CPF members already suggested that they might act as photolyase. This was further confirmed by in vitro repair studies (Fig. 7). Cry‐DASH proteins are known to repair CPDs specifically in single‐stranded DNA (Selby and Sancar 2006; Pokorny et al. 2008). Both cry‐DASHs repaired these lesions in a light‐dependent fashion to similar extent (Fig. 7A, B). Cry‐DASHs have been described in other fungi, and for some of them, a minor sensory role was found. For example, they participate in the light regulation of development in *S. sclerotiorum* and *F. fujikuroi* (Veluchamy and Rollins 2008; Castrillo et al. 2013), and of pigment accumulation in *F. fujikuroi* (Castrillo et al. 2013). The photolyase activity of Cry1 and Cry2, described here does not preclude a sensory function of these proteins. Most genes identified in our study as blue light controlled are under control of Wco1, but a few still responded to blue light in the *Δwco1* mutant (Table 1). Future studies needs to address which of the photoreceptors including Cry1 and Cry2, regulate induction of light‐controlled genes so far unidentified ones from our transcriptome studies.

The DNA repair assays with Phr1 were performed with CPDs present in ssDNA and dsDNA. In contrast to cry‐DASH, canonical CPD‐photolyases repair these lesions in both, ssDNA and dsDNA (Sancar 2003). As expected from its sequence homology with canonical class I CPD‐photolyases, Phr1 repaired CPDs in both substrates (Fig. 7 C, D). This is corroborated by our observation that *Δphr1* mutants are deficient in photoreactivation (Fig. 6). In addition, we observed repair of (6–4)‐photoproducts by Phr2 (Fig. 7E) demonstrating that it is indeed a functional (6–4)‐photolyase. We did not test whether Phr2 repairs CPDs and whether Phr1 repairs (6–4)‐photoproducts because photolyases are known to be very specific for either one of these substrates (Sancar 2003).

Wco1‐dependent induction of *phr1* and *phr2* suggested that a *Δwco1* mutant might be less resistant to UV‐B due to lower levels of photolyase. Therefore, we tested photoreactivation in *Δwco1* cells and found indeed a much lower rate of survivors compared to wild‐type (Fig. 8). Nevertheless, *Δwco1* cells still showed a positive effect of visible light on survival compared to the *Δphr1Δphr2* double mutant. This is most likely due to the residual induction of *phr1* in the Δ*wco1* mutant (Table. 1, Fig. 4). These data also demonstrate that Wco1 is important for *U. maydis* to sense adverse environmental conditions including such of elevated UV‐exposure. Such a role is not unique to *U. maydis* since deletion of *white collar 1* in other fungi like the basidiomycete *C. neoformans* have been found to cause UV‐sensitivity, too (Idnurm and Heitman 2005; Verma and Idnurm 2013). Moreover, induction of DNA repair enzymes by visible light has been shown for other fungal species such as the ascomycetes *N. crassa, A. fulmigatus* (Fuller et al. 2013), *A. nidulans*, (Ruger‐Herreros et al. 2011), *F. oxysporum* (Ruiz‐Roldan et al. 2008), and *C. zea‐maydis* (Yu et al. 2013).

With this study, we aimed to lay the basis for the photobiology of *U. maydis*. Further investigations as to how light affect downstream signaling cascades in *U. maydis* especially with respect to its interaction with the host plant maize, will be of particular interest.

## Conflict of Interest

None declared.

## Supporting information


**Figure S1.** Spectrum of UV‐B source used in photoreactivation experiments.
**Figure S2.** Purification and absorption spectra of *U. maydis* CPF members
**Figure S3**. Light–dark difference spectra of *U. maydis* CPF members.
**Table S1.** *U. maydis* strains used in this study.
**Table S2**. Primers used for creating *U. maydis* deletion strains.
**Table S3.** Primers used for qRT‐PCR and gene cloning.
**Table S4.** *E. coli* strains used in this study.
**Table S5.** Primers used in this study for *E. coli* expression constructs.
**Table S6.** Probes used for in vitro repair assays.

## References

[mbo3322-bib-0001] Avalos, J. , and A. F. Estrada . 2010 Regulation by light in Fusarium. Fungal Genet. Biol. 47:930–938. 20460165 10.1016/j.fgb.2010.05.001

[mbo3322-bib-0002] Bailey, T. L. , and C. Elkan . 1994 Fitting a mixture model by expectation maximization to discover motifs in biopolymers. Proc. Natl. Int. Conf. Intell. Syst. Mol. Biol. 2:28–36. 7584402

[mbo3322-bib-0003] Ballario, P. , and G. Macino . 1997 White collar proteins: PASsensing the light signal in *Neurospora crassa* . Trends Microbiol. 5:458–462. 9402704 10.1016/S0966-842X(97)01144-X

[mbo3322-bib-0004] Ballario, P. , P. Vittorioso , A. Magrelli , C. Talora , A. Cabibbo , and G. Macino . 1996 White collar 1, a central regulator of blue light responses in Neurospora, is zinc finger protein. EMBO J. 15:1650–1657. 8612589 PMC450076

[mbo3322-bib-0005] Banuett, F. 1995 Genetics of *Ustilago maydis*, a fungal pathogen that induces tumors in maize. Annu. Rev. Genetics 29:179–208. 8825473 10.1146/annurev.ge.29.120195.001143

[mbo3322-bib-0006] Banuett, F. , and I. Herskowitz . 1989 Different a alleles of *Ustilago maydis* are necessary for maintenance of filamentous growth but not for meiosis. Proc. Natl Acad. Sci. USA 86:5878–5882. 16594058 10.1073/pnas.86.15.5878 PMC297734

[mbo3322-bib-0009] Bayram, Ö. , C. Biesemann , S. Krappmann , P. Galland , and G. H. Braus . 2008 More than a repair enzyme: *Aspergillus nidulans* photolyase‐like CryA is a regulator of sexual development. Mol. Biol. Cell 19:3254–3262. 18495868 10.1091/mbc.E08-01-0061 PMC2488289

[mbo3322-bib-0010] Bayram, Ö. , G. H. Braus , R. Fischer , and J. Rodriguez‐Romero . 2010 Spotlights on *Aspergillus nidulans* photosensory systems. Fungal Genet. Biol. 47:900–908. 20573560 10.1016/j.fgb.2010.05.008

[mbo3322-bib-0011] Berrocal‐Tito, G. M. , E. U. Esquivel‐Naranjo , B. A. Horwitz , and A. Herrera‐Estrella . 2007 *Trichoderma atroviride* PHR1, a fungal photolyase responsible for DNA repair, autoregulates its own photoinduction. Eukaryot. Cell 6:1682–1692. 17545314 10.1128/EC.00208-06 PMC2043357

[mbo3322-bib-0012] Bluhm, B. H. , and L. D. Dunkle . 2008 PHL1 of *Cercospora zeae‐maydis* encodes a member of the photolyase/cryptochrome family involved in UV protection and fungal development. Fungal Genet. Biol. 45:1364–1372. 18682297 10.1016/j.fgb.2008.07.005

[mbo3322-bib-0013] Blumenstein, A. , K. Vienken , R. Tasler , J. Purschwitz , D. Veith , N. Frankenberg‐Dinkel , et al. 2005 The *Aspergillus nidulans* phytochrome FphA represses sexual development in red light. Curr. Biol. 15:1833–1838. 16243030 10.1016/j.cub.2005.08.061

[mbo3322-bib-0014] Böhmer, C. , M. Böhmer , M. Bölker , and B. Sandrock . 2008 Cdc42 and the Ste20‐like kinase Don3 act independently in triggering cytokinesis in *Ustilago maydis* . J. Cell Sci. 121:143–148. 18089648 10.1242/jcs.014449

[mbo3322-bib-0015] Brachmann, A. , G. Weinzierl , J. Kämper , and R. Kahmann . 2001 Identification of genes in the bW/bE regulatory cascade in *Ustilago maydis* . Mol. Microbiol. 42:1047–1063. 11737646 10.1046/j.1365-2958.2001.02699.x

[mbo3322-bib-0016] Brachmann, A. , J. König , C. Julius , and M. Feldbrügge . 2004 A reverse genetic approach for generating gene replacement mutants in *Ustilago maydis* . Mol. Genet. Genomics 272:216–226. 15316769 10.1007/s00438-004-1047-z

[mbo3322-bib-0017] Braga, G. U. L. , D. E. N. Rangel , É. K. K. Fernandes , S. D. Flint , and D. W. Roberts . 2015 Molecular and physiological effects of environmental UV radiation on fungal conidia. Curr. Genet. 61:405–425. 25824285 10.1007/s00294-015-0483-0

[mbo3322-bib-0018] Brandt, S. , D. von Stetten , M. Günther , P. Hildebrandt , and N. Frankenberg‐Dinkel . 2008 The fungal phytochrome FphA from *Aspergillus nidulans* . J. Biol. Chem. 283:34605–34614. 18931394 10.1074/jbc.M805506200 PMC3259886

[mbo3322-bib-0019] Braus, G. H. , S. Irniger , and Ö. Bayram . 2010 Fungal development and the COP9 signalosome. Curr. Opin. Microbiol. 13:672–676. 20934903 10.1016/j.mib.2010.09.011

[mbo3322-bib-0020] Brefort, T. , G. Doehlemann , A. Mendoza‐Mendoza , S. Reissmann , A. Djamei , and R. Kahmann . 2009 *Ustilago maydis* as a pathogen. Annu. Rev. Phytopathol. 47:423–445. 19400641 10.1146/annurev-phyto-080508-081923

[mbo3322-bib-0021] Briggs W. R., and Spudich J. L., eds. 2005 Handbook of Photosensory Receptors. Wiley‐VCH, Weinheim, Germany.

[mbo3322-bib-0022] Brudler, R. , K. Hitomi , H. Daiyasu , H. Toh , K. Kucho , M. Ishiura , et al. 2003 Identification of a new cryptochrome class: structure, function, and evolution. Mol. Cell 11:59–67. 12535521 10.1016/s1097-2765(03)00008-x

[mbo3322-bib-0023] Cadet, J. , and R. Wagner . 2013 DNA base damage by reactive oxygen species, oxidizing agents, and UV radiation. Cold Spring Harb. Perspect. Biol. 2013:a012559. 23378590 10.1101/cshperspect.a012559 PMC3552502

[mbo3322-bib-0024] Castrillo, M. , J. García‐Martínez , and J. Avalos . 2013 Light‐dependent functions of the *Fusarium fujikuroi* CryD DASH cryptochrome in development and secondary metabolism. Appl. Environ. Microbiol. 79:2777–2788. 23417004 10.1128/AEM.03110-12 PMC3623198

[mbo3322-bib-0025] Chaves, I. , R. Pokorny , M. Byrdin , N. Hoang , T. Ritz , K. Brettel , et al. 2011 The cryptochromes: blue light photoreceptors in plants and animals. Annu. Rev. Plant Biol. 62:335–364. 21526969 10.1146/annurev-arplant-042110-103759

[mbo3322-bib-0026] Chen, C. H. , C. S. Ringelberg , R. H. Gross , J. C. Dunlap , and J. J. Loros . 2009 Genome‐wide analysis of light‐inducible responses reveals hierarchical light signalling in *Neurospora* . EMBO J. 28:1029–1042. 19262566 10.1038/emboj.2009.54 PMC2683703

[mbo3322-bib-0027] Chen, C. H. , J. C. Dunlap , and J. J. Loros . 2010 Neurospora illuminates fungal photoreception. Fungal Genet. Biol. 47:922–929. 20637887 10.1016/j.fgb.2010.07.005 PMC3649881

[mbo3322-bib-0028] Christensen, J. J. 1963 Corn smut caused by *Ustilago maydis* . Am. Phytopathol. Soc. Monogr. 2:1–41.

[mbo3322-bib-0030] Corrochano, L. M. , and V. Garre . 2010 Photobiology in the Zygomycota: multiple photoreceptor genes for complex responses to light. Fungal Genet. Biol. 47:893–899. 20466063 10.1016/j.fgb.2010.04.007

[mbo3322-bib-0031] Crosthwaite, S. K. , J. C. Dunlap , and J. J. Loros . 1997 *Neurospora* wc‐1 and wc‐2: transcription, photoresponses, and the origins of circadian rhythmicity. Science 276:763–769. 9115195 10.1126/science.276.5313.763

[mbo3322-bib-0032] Degli‐Innocenti, F. , J. A. Chambers , and V. E. Russo . 1984 Conidia induce the formation of protoperithecia in *Neurospora crassa*: further characterization of white collar mutants. J. Bacteriol. 159:808–810. 6235212 10.1128/jb.159.2.808-810.1984 PMC215725

[mbo3322-bib-0033] Djamei, A. , and R. Kahmann . 2012 *Ustilago maydis*: dissecting the molecular interface between pathogen and plant. PLoS Pathog. 8:e1002955. doi:10.1371/journal.ppat.1002955. 23133380 10.1371/journal.ppat.1002955 PMC3486881

[mbo3322-bib-0034] Eichhorn, H. , F. Lessing , B. Winterberg , J. Schirawski , J. Kämper , P. Müller , et al. 2006 A ferroxidation/permeation iron uptake system is required for virulence in *Ustilago maydis* . Plant Cell 18:3332–3345. 17138696 10.1105/tpc.106.043588 PMC1693961

[mbo3322-bib-0035] Essen, L.‐O. 2006 Photolyases and cryptochromes: common mechanisms of DNA repair and light‐driven signaling? Curr. Opin. Struct. Biol. 16:51–59. 16427270 10.1016/j.sbi.2006.01.004

[mbo3322-bib-0036] Estrada, A. F. , T. Brefort , C. Mengel , V. Diaz‐Sanchez , A. Alder , S. Al‐Babili , et al. 2009 *Ustilago maydis* accumulates *β*‐carotene at levels determined by a retinal‐forming carotenoid oxygenase. Fungal Genet. Biol. 46:803–813. 19584000 10.1016/j.fgb.2009.06.011

[mbo3322-bib-0037] Freitag, J. , D. Lanver , C. Böhmer , K. O. Schink , M. Bölker , and B. Sandrock . 2011 Septation of infectious hyphae is critical for appressoria formation and virulence in the smut fungus *Ustilago maydis* . PLoS Pathog. 7:e1002044. doi:10.1371/journal.ppat.1002044. 21625538 10.1371/journal.ppat.1002044 PMC3098242

[mbo3322-bib-0038] Froehlich, A. C. , Y. Liu , J. J. Loros , and J. C. Dunlap . 2002 White collar‐1, a circadian blue light photoreceptor, binding to the frequency promoter. Science 297:815–819. 12098706 10.1126/science.1073681

[mbo3322-bib-0039] Froehlich, A. C. , C. H. Chen , W. J. Belden , C. Madeti , T. Roenneberg , M. Merrow , et al. 2010 Genetic and molecular characterization of a cryptochrome from the filamentous fu ngus *Neurospora crassa* . Eukaryot. Cell 9:738–750. 20305004 10.1128/EC.00380-09 PMC2863965

[mbo3322-bib-0040] Fuller, K. K. , C. S. Ringelberg , J. J. Loros , and J. C. Dunlap . 2013 The fungal pathogen *Aspergillus fumigatus* regulates growth, metabolism, and stress resistance in response to light. MBio 4: pii E00142–13. 23532976 10.1128/mBio.00142-13 PMC3604765

[mbo3322-bib-0041] Fuller, K. K. , J. J. Loros , and J. C. Dunlap . 2015 Fungal photobiology: visible light as a signal for stress, space and time. Curr. Genet. 61:275–288. 25323429 10.1007/s00294-014-0451-0 PMC4401583

[mbo3322-bib-0042] Garzia‐Martinez, J. , M. Brunk , J. Avalos , and U. Terpitz . 2015 The CarO rhodopsin of the fungus *Fusarium fujikuroi* is a light‐driven proton pump that retards spore germination. Sci. Rep. 5:7798. doi:10.1028/srep07798. 25589426 10.1038/srep07798 PMC4295100

[mbo3322-bib-0043] Geisselbrecht, Y. , S. Frühwirth , C. Schroeder , A. J. Pierik , G. Klug , and L.‐O. Essen . 2012 CryB from *Rhodobacter sphaeroides*: a unique class of cryptochromes with new cofactors. EMBO Rep. 13:223–229. 22290493 10.1038/embor.2012.2 PMC3323124

[mbo3322-bib-0044] Ghosh, A. 2014 Small heat shock proteins (HSP12, HSP20 and HSP30) play a role in *Ustilago maydis* pathogenesis. FEMS Microbiol. Lett. 361:17–24. 10.1111/1574-6968.12605 25251081

[mbo3322-bib-0045] Hartmann, H. A. , J. Krüger , F. Lottspeich , and R. Kahmann . 1999 Environmental signals controlling sexual development of the corn smut fungus *Ustilago maydis* through the transcriptional regulator Prf1. Plant Cell 11:1293–1305. 10402430 10.1105/tpc.11.7.1293 PMC144278

[mbo3322-bib-0046] He, Q. , and Y. Liu . 2005 Molecular mechanism of the light response in Neurospora: from light‐induced transcription to photoadaptation. Genes Dev. 19:2888–2899. 16287715 10.1101/gad.1369605 PMC1315395

[mbo3322-bib-0047] He, Q. , P. Cheng , Y. H. Yang , L. Wang , K. H. Gardner , and Y. Liu . 2002 White collar‐1, a DNA binding transcription factor and a light sensor. Science 297:840–843. 12098705 10.1126/science.1072795

[mbo3322-bib-0048] Heimel, K. , M. Scherer , D. Schuler , and J. Kämper . 2010 The *Ustilago maydis* Clp1 protein orchestrates pheromone and b‐dependent signaling pathways to coordinate the cell cycle and pathogenic development. Plant Cell 22:2908–2922. 20729384 10.1105/tpc.110.076265 PMC2947178

[mbo3322-bib-0049] Herrera‐Estrella, A. , and B. A. Horwitz . 2007 Looking through the eyes of fungi: molecular genetics of photoreception. Mol. Microbiol. 64:5–15. 17376067 10.1111/j.1365-2958.2007.05632.x

[mbo3322-bib-0050] Hoffman, C. S. , and F. Winston . 1987 A 10‐minute DNA preparation from yeast efficiently releases autonomous plasmids for transformation of *Escherichia coli* . Gene 57:267–272. 3319781 10.1016/0378-1119(87)90131-4

[mbo3322-bib-0051] Holloman, W. K. , J. Schirawski , and R. Holliday . 2007 Towards understanding the extreme radiation resistance of *Ustilago maydis* . Trends Microbiol. 15:525–529. 17997098 10.1016/j.tim.2007.10.007

[mbo3322-bib-0052] Holloman, W. K. , J. Schirawski , and R. Holliday . 2008 The homologous recombination system of *Ustilago maydis* . Fungal Genet. Biol. 45(Suppl. 1):S31–S39. 18502156 10.1016/j.fgb.2008.04.006 PMC2583931

[mbo3322-bib-0053] Hothorn, M. , T. Dabi , and J. Chory . 2011 Structural basis for cytokinin recognition by *Arabidopsis thaliana* histidine kinase 4. Nat. Chem. Biol. 7:766–768. 21964459 10.1038/nchembio.667 PMC3197759

[mbo3322-bib-0054] Huala, E. , P. W. Oeller , E. Liscum , I.‐S. Han , E. Larsen , and W. R. Briggs . 1997 *Arabidopsis* NPH1: a protein kinase with a putative redox‐sensing domain. Science 278:2120–2123. 9405347 10.1126/science.278.5346.2120

[mbo3322-bib-0055] Idnurm, A. , and J. Heitman . 2005 Light controls growth and development via a conserved pathway in the fungal kingdom. PLoS Biol. 3:615–626. 10.1371/journal.pbio.0030095 PMC1064852 15760278

[mbo3322-bib-0056] Idnurm, A. , and J. Heitman . 2010 Ferrochelatase is a conserved downstream target of the blue light‐sensing White collar complex in fungi. Microbiology 156:2393–2407. 20488877 10.1099/mic.0.039222-0 PMC3068673

[mbo3322-bib-0057] Idnurm, A. , S. Verma , and L. M. Corrochano . 2010 A glimpse into the basis of vision in the kingdom *Mycota* . Fungal Genet. Biol. 47:881–892. 20451644 10.1016/j.fgb.2010.04.009 PMC2950209

[mbo3322-bib-0058] Iseki, M. , S. Matsunaga , A. Murakami , K. Ohno , K. Shiga , K. Yoshida , et al. 2002 A blue‐light‐activated adenylyl cyclase mediates photoavoidance in *Euglena gracilis* . Nature 415:1047–1051. 11875575 10.1038/4151047a

[mbo3322-bib-0059] Kahmann, R. , G. Steinberg , C. Basse , M. Feldbrügge , and J. Kämper . 2000 *Ustilago maydis*, the causative agent of corn smut disease Pp. 347–371 *in* Kronstad J. W., ed. Fungal Pathology. Kluwer Academic Publisher, Dordrecht, The Netherlands.

[mbo3322-bib-0060] Kaldi, K. , B. H. Gonzalez , and M. Brunner . 2006 Transcriptional regulation of the Neurospora circadian clock gene wc‐1 affects the phase of circadian output. EMBO Rep. 7:199–204. 16374510 10.1038/sj.embor.7400595 PMC1369249

[mbo3322-bib-0061] Kamada, T. , H. Sano , T. Nakazawa , and K. Nakahori . 2010 Regulation of fruiting body photomorphogenesis in *Coprinopsis cinerea* . Fungal Genet. Biol. 47:917–921. 20471485 10.1016/j.fgb.2010.05.003

[mbo3322-bib-0062] Kämper, J. 2004 A PCR‐based system for highly efficient generation of gene replacement mutants in *Ustilago maydis* . Mol. Gen. Genomics 271:103–110. 10.1007/s00438-003-0962-8 14673645

[mbo3322-bib-0063] Kämper, J. , R. Kahmann , M. Bölker , L. J. Ma , T. Brefort , B. J. Saville , et al. 2006 Insights from the genome of the biotrophic fungal plant pathogen *Ustilago maydis* . Nature 444:97–101. 17080091 10.1038/nature05248

[mbo3322-bib-0064] Kangatharalingam, N. , and M. W. Ferguson . 1984 A simple and rapid technique for fluorescence staining of fungal nuclei. Curr. Microbiol. 10:99–104.

[mbo3322-bib-0065] Kelner, A. 1949 Effect of visible light on the recovery of *Streptomyces griseus* conidia from ultraviolet irradiation injury. Proc. Natl Acad. Sci. USA 35:73–79. 16588862 10.1073/pnas.35.2.73 PMC1062964

[mbo3322-bib-0066] Kim, S. T. , and A. Sancar . 1991 Effect of base, pentose, and phosphodiester backbone structures on binding and repair of pyrimidine dimers by *Escherichia coli* DNA photolyase. Biochemistry 30:8623–8630. 1716150 10.1021/bi00099a019

[mbo3322-bib-0067] Kiontke, S. , Y. Geisselbrecht , R. Pokorny , T. Carell , A. Batschauer , and L.‐O. Essen . 2011 Crystal structures of an archaeal class II DNA photolyase and its complex with UV‐damaged duplex DNA. EMBO J. 30:4437–4449. 21892138 10.1038/emboj.2011.313 PMC3230371

[mbo3322-bib-0068] Klar, T. , R. Pokorny , J. Moldt , A. Batschauer , and L.‐O. Essen . 2007 Cryptochrome 3 from *Arabidopsis thaliana*: structural and functional analysis of its complex with a folate light antenna. J. Mol. Biol. 366:954–964. 17188299 10.1016/j.jmb.2006.11.066

[mbo3322-bib-0069] Kleine, T. , P. Lockhart , and A. Batschauer . 2003 An *Arabidopsis* protein closely related to *Synechocystis* cryptochrome is targeted to organelles. Plant J. 35:93–103. 12834405 10.1046/j.1365-313x.2003.01787.x

[mbo3322-bib-0070] Kuratani, M. , K. Tanaka , K. Terashima , H. Muraguchi , T. Nakazawa , K. Nakahori , et al. 2010 The dst2 gene essential for photomorphogenesis of *Coprinopsis cinerea* encodes a protein with a putative FAD‐binding‐4 domain. Fungal Genet. Biol. 47:152–158. 19850145 10.1016/j.fgb.2009.10.006

[mbo3322-bib-0071] Lewis, Z. A. , A. Correa , C. Schwerdtfeger , K. L. Link , X. Xie , R. H. Gomer , et al. 2002 Overexpression of White Collar‐1 (WC‐1) activates circadian clock‐associated genes, but is not sufficient to induce most light‐regulated gene expression in *Neurospora crassa* . Mol. Microbiol. 45:917–931. 12180913 10.1046/j.1365-2958.2002.03074.x

[mbo3322-bib-0072] Linden, H. , and G. Macino . 1997 White collar 2, a partner in blue‐light signal transduction, controlling expression of light‐regulated genes in *Neurospora crassa* . EMBO J. 16:98–109. 9009271 10.1093/emboj/16.1.98 PMC1169617

[mbo3322-bib-0073] Linden, H. , P. Ballario , and G. Macino . 1997 Blue light regulation in *Neurospora crassa* . Fungal Genet. Biol. 22:141–150. 9454641 10.1006/fgbi.1997.1013

[mbo3322-bib-0074] Losi, A. , and W. Gärtner . 2012 The evolution of flavin‐binding photoreceptors: an ancient chromophore serving trendy blue‐light sensors. Annu. Rev. Plant Biol. 63:49–72. 22136567 10.1146/annurev-arplant-042811-105538

[mbo3322-bib-0075] Mahlert, M. , L. Leveleki , B. Hlubek , B. Sandrock , and M. Bölker . 2006 Rac1 and Cdc42 regulate hyphal growth and cytokinesis in the dimorphic fungus *Ustilago maydis* . Mol. Microbiol. 59:567–578. 16390450 10.1111/j.1365-2958.2005.04952.x

[mbo3322-bib-0076] Möckli, N. , and D. Auerbach . 2004 Quantitative beta‐galactosidase assay suitable for high‐throughput applications in the yeast two‐hybrid system. Biotechniques 36:872–876. 15152608 10.2144/04365PT03

[mbo3322-bib-0077] Müller, M. , and T. Carell . 2009 Structural biology of DNA photolyases and cryptochromes. Curr. Opin. Struct. Biol. 19:1–9. 19487120 10.1016/j.sbi.2009.05.003

[mbo3322-bib-0078] Nelson, M. A. , G. Morelli , A. Carattoli , N. Romano , and G. Macino . 1989 Molecular cloning of a *Neurospora crassa* caroteinoid biosynthetic gene (*albino‐3*) regulated by blue light and the products of the white collar genes. Mol. Cell. Biol. 9:1271–1276. 2524647 10.1128/mcb.9.3.1271-1276.1989 PMC362718

[mbo3322-bib-0079] Nsa, I. Y. , N. Karunarathna , X. Liu , H. Huang , B. Boetteger , and D. Bell‐Pedersen . 2015 A novel cryptochrome‐dependent oscillator in *Neurospora crassa* . Genetics 199:233–245. 25361899 10.1534/genetics.114.169441 PMC4286687

[mbo3322-bib-0080] Oberpichler, I. , A. J. Pierik , J. Wesslowski , R. Pokorny , R. Rosen , M. Vugman , et al. 2011 A photolyase‐like protein from Agrobacterium tumefaciens with an iron‐sulfur cluster. PLoS ONE 6:e26775. doi:10.1371/journal.pone.0026775. 22066008 10.1371/journal.pone.0026775 PMC3204975

[mbo3322-bib-0081] Olmedo, M. , C. Ruger‐Herreros , E. M. Luque , and L. M. Corrochano . 2010 A complex photoreceptor system mediates the regulation by light of the conidiation genes con‐10 and con‐6 in *Neurospora crassa* . Fungal Genet. Biol. 47:352–363. 19932184 10.1016/j.fgb.2009.11.004

[mbo3322-bib-0082] Pokorny, R. , T. Klar , L. O. Essen , and A. Batschauer . 2005 Crystallization and preliminary X‐ray analysis of cryptochrome 3 from *Arabidopsis thaliana* . Acta Cryst. F61:935–938. 10.1107/S1744309105028897 PMC1991327 16511200

[mbo3322-bib-0083] Pokorny, R. , T. Klar , U. Hennecke , T. Carell , A. Batschauer , and L.‐O. Essen . 2008 Recognition and repair of UV‐lesions in loop structures of duplex DNA by DASH‐type cryptochrome. Proc. Natl Acad. Sci. USA 105:21023–21027. 19074258 10.1073/pnas.0805830106 PMC2634942

[mbo3322-bib-0084] Purschwitz, J. , S. Müller , C. Kastner , M. Schöser , H. Haas , E. A. Espeso , et al. 2008 Functional and physical interaction of blue‐ and red‐light sensors in *Aspergillus nidulans* . Curr. Biol. 18:255–259. 18291652 10.1016/j.cub.2008.01.061

[mbo3322-bib-0086] Resnick, M. A. , and R. Holliday . 1971 Genetic repair and the synthesis of nitrate reductase in *Ustilago maydis* after UV irradiation. Mol. Gen. Genetics 111:171–184.

[mbo3322-bib-0087] Rodriguez‐Romero, J. , M. Hedtke , C. Kastner , S. Müller , and R. Fischer . 2010 Fungi, hidden in soil or up in the air: light makes a difference. Annu. Rev. Microbiol. 64:585–610. 20533875 10.1146/annurev.micro.112408.134000

[mbo3322-bib-0088] Ruger‐Herreros, C. , L. Rodriguez‐Romero , R. Fernández‐Barranco , M. Olmedo , R. Fischer , L. M. Corrochano , et al. 2011 Regulation of conidiation by light in *Aspergillus nidulans* . Genetics 188:809–822. 21624998 10.1534/genetics.111.130096 PMC3176103

[mbo3322-bib-0089] Ruiz‐Roldan, M. C. , V. Garre , J. Guarro , M. Marine , and M. I. Roncero . 2008 Role of White Collar 1 photoreceptor in carotenogenesis, UV resistance, hydrophobicity, and virulence of *Fusarium oxysporum* . Eukaroyt. Cell 7:1227–1230. 10.1128/EC.00072-08 PMC2446679 18503005

[mbo3322-bib-0090] Sambrook, J. , E. F. Fritsch , and T. Maniatis . 1989 Molecular cloning: a laboratory manual. Cold Spring Harbor Laboratory, Cold Spring Harbor, New York.

[mbo3322-bib-0091] Sancar, A. 2003 Structure and function of DNA photolyase and cryptochrome blue‐light photoreceptors. Chem. Rev. 103:2203–2237. 12797829 10.1021/cr0204348

[mbo3322-bib-0092] Schmoll, M. , E. U. Esquivel‐Naranjo , and A. Herrera‐Estrella . 2010 *Trichoderma* in the light of day – physiology and development. Fungal Genet. Biol. 47:909–916. 20466064 10.1016/j.fgb.2010.04.010 PMC2954361

[mbo3322-bib-0093] Schulz, B. , F. Banuett , M. Dahl , R. Schlesinger , W. Schafer , T. Martin , et al. 1990 The b‐alleles of *U. maydis*, whose combinations program pathogenic development, code for polypeptides containing a homeodomain‐related motif. Cell 60:295–306. 1967554 10.1016/0092-8674(90)90744-y

[mbo3322-bib-0094] Schuster, M. , R. Lipowsky , M. A. Assmann , P. Lenz , and G. Steinberg . 2011 Transient binding of dynein controls bidirectional long‐range motility of early endosomes. Proc. Natl Acad. Sci. USA 108:3618–3623. 21317367 10.1073/pnas.1015839108 PMC3048114

[mbo3322-bib-0095] Schwerdtfeger, C. , and H. Linden . 2003 VIVID is a flavoprotein and serves as a fungal blue light photoreceptor for photoadaptation. EMBO J. 22:4846–4855. 12970196 10.1093/emboj/cdg451 PMC212719

[mbo3322-bib-0096] Selby, C. P. , and A. Sancar . 2006 A cryptochrome/photolyase class of enzymes with single‐stranded DNA‐specific photolyase activity. Proc. Natl Acad. Sci. USA 103:17696–17700. 17062752 10.1073/pnas.0607993103 PMC1621107

[mbo3322-bib-0097] Shrode, L. B. , Z. A. Lewis , L. D. White , D. Bell‐Pedersen , and D. J. Ebbole . 2001 vvd is required for light adaptation of conidiation‐specific genes of *Neurospora crassa*, but not circadian conidiation. Fungal Genet. Biol. 32:169–181. 11343403 10.1006/fgbi.2001.1264

[mbo3322-bib-0098] Steinberg, G. , and J. Perez‐Martin . 2008 *Ustilago maydis*, a new fungal model system for cell biology. Trends Cell Biol. 18:61–67. 18243705 10.1016/j.tcb.2007.11.008

[mbo3322-bib-0099] Swartz, T. E. , and R. A. Bogomolni . 2005 LOV‐domain photochemistry Pp. 305–321 *in* Briggs W. R. and Spudich J. L., eds. Handbook of photosensory receptors. Wiley‐VCH, Weinheim, Germany.

[mbo3322-bib-0100] Tsukuda, T. , S. Carleton , S. Fotheringham , and W. K. Holloman . 1988 Isolation and characterization of an autonomously replicating sequence from *Ustilago maydis* . Mol. Cell. Biol. 8:3703–3709. 2851726 10.1128/mcb.8.9.3703-3709.1988 PMC365426

[mbo3322-bib-0101] Veluchamy, S. , and J. A. Rollins . 2008 A CRY‐DASH‐type photolyase/cryptochrome from *Sclerotinia sclerotiorum* mediates minor UV‐A‐specific effects on development. Fungal Genet. Biol. 45:1265–1276. 18644246 10.1016/j.fgb.2008.06.004

[mbo3322-bib-0102] Verma, S. , and A. Idnurm . 2013 The Uve1 endonuclease is regulated by the white collar complex to protect *Cryptococcus neoformans* from UV damage. PLoS Genet. 9:e1003769. doi:10.1371/journal.pgen.1003769. 24039606 10.1371/journal.pgen.1003769 PMC3764193

[mbo3322-bib-0103] Vollmeister, E. , K. Schipper , S. Baumann , C. Haag , T. Pohlmann , J. Stock , et al. 2011 Fungal development of the plant pathogen *Ustilago maydis* . FEMS Microbiol. Rev. 36:59–77. 21729109 10.1111/j.1574-6976.2011.00296.x

[mbo3322-bib-0104] Yamamoto, J. , R. Martin , S. Iwai , P. Plaza , and K. Brettel . 2013 Repair of the (6‐4) photoproduct by DNA photolyase requires two photons. Angew. Chem. Int. Ed. 52:7432–7436. 10.1002/anie.201301567 23761226

[mbo3322-bib-0105] Yu, S. M. , G. Ramkumar , and Y. H. Lee . 2013 Light quality influences the virulence and physiological responses of *Colletotrichum acutatum* causing anthracnose in pepper plants. J. Appl. Microbiol. 115:509–516. 23663215 10.1111/jam.12252

[mbo3322-bib-0106] Zhang, F. , P. Scheerer , I. Oberpichler , T. Lamparter , and N. Krauss . 2013 Crystal structure of a prokaryotic (6‐4) photolyase with an Fe‐S cluster and a 6,7‐dimethyl‐8‐ribityllumazine antenna chromophore. Proc. Natl Acad. Sci. USA 110:7217–7222. 23589886 10.1073/pnas.1302377110 PMC3645588

[mbo3322-bib-0107] Zirak, P. , A. Penzkofer , J. Moldt , R. Pokorny , A. Batschauer , and L.‐O. Essen . 2009 Photocycle dynamics of the E149A mutant of cryptochrome 3 from *Arabidopsis thaliana* . J. Photochem. Photobiol. B: Biol. 97:94–108. 10.1016/j.jphotobiol.2009.08.005 19800811

