# Supplementary material for: White collar 1‐induced photolyase expression contributes to UV‐tolerance of Ustilago maydis
Source: Microbiologyopen. 2015 Dec 20;5(2):224–43. doi: 10.1002/mbo3.322 (PMC4831468; doi:10.1002/mbo3.322)
Supplement: Supplementary file 1 — Figure S1. Spectrum of UV‐B source used in photoreactivation experiments. Figure S2. Purification and absorption spectra of U. maydis CPF members Figure S3. Light–dark difference spectra of U. maydis CPF members. Table S1. U. maydis strains used in this study. Table S2. Primers used for creating U. maydis deletion strains. Table S3. Primers used for qRT‐PCR and gene cloning. Table S4. E. coli strains used in this study. Table S5. Primers used in this study for E. coli expression constructs. Table S6. Probes used for in vitro repair assays. [file MBO3-5-224-s001.pdf]

Supplementary Material for:

White collar 1-induced photolyase expression contributes to UV-tolerance of *Ustilago maydis*

Annika Brych, Judita Mascarenhas, Elaine Jaeger, Elzbieta Charkiewicz, Richard Pokorny, Michael Bölker, Gunther Doehlemann & Alfred Batschauer

Includes Figures S1-S3 and Tables S1-S6

Fig. S1. Spectrum of UV-B source used in photoreactivation experiments.

Fig. S2. Purification and absorption spectra of *U. maydis* CPF members.

Fig. S3. Light-dark difference spectra of *U. maydis* CPF members.

Tab. S1. *U. maydis* strains used in this study.

Tab. S2. Primers used for creating *U. maydis* deletion strains.

Tab. S3. Primers used for qRT-PCR and gene cloning.

Tab. S4. *E. coli* strains used in this study.

Tab. S5. Primers used in this study to create *E. coli* expression constructs.

Tab. S6. Probes used for *in vitro* repair assays.

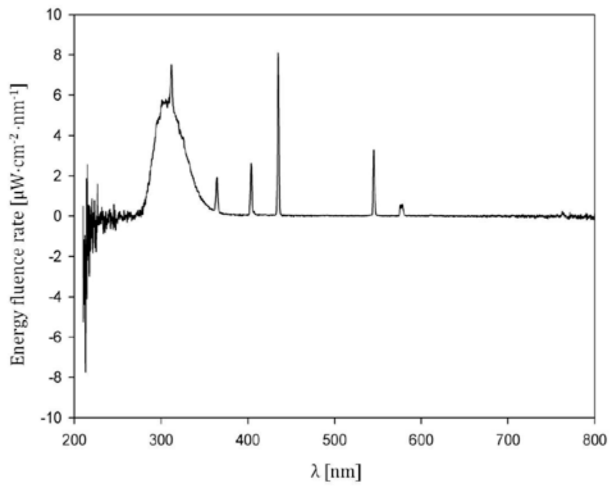

Fig. S1. Spectrum of UV-B source used in photoreactivation experiments. Used were UV-B tubes (TL40W12RS, Philips) wrapped with one layer of cellulose acetate filter (Diacel West Design). The energy fluence rate in the UV-B region (280 nm – 320 nm) was 1.83 W m^{-2}.

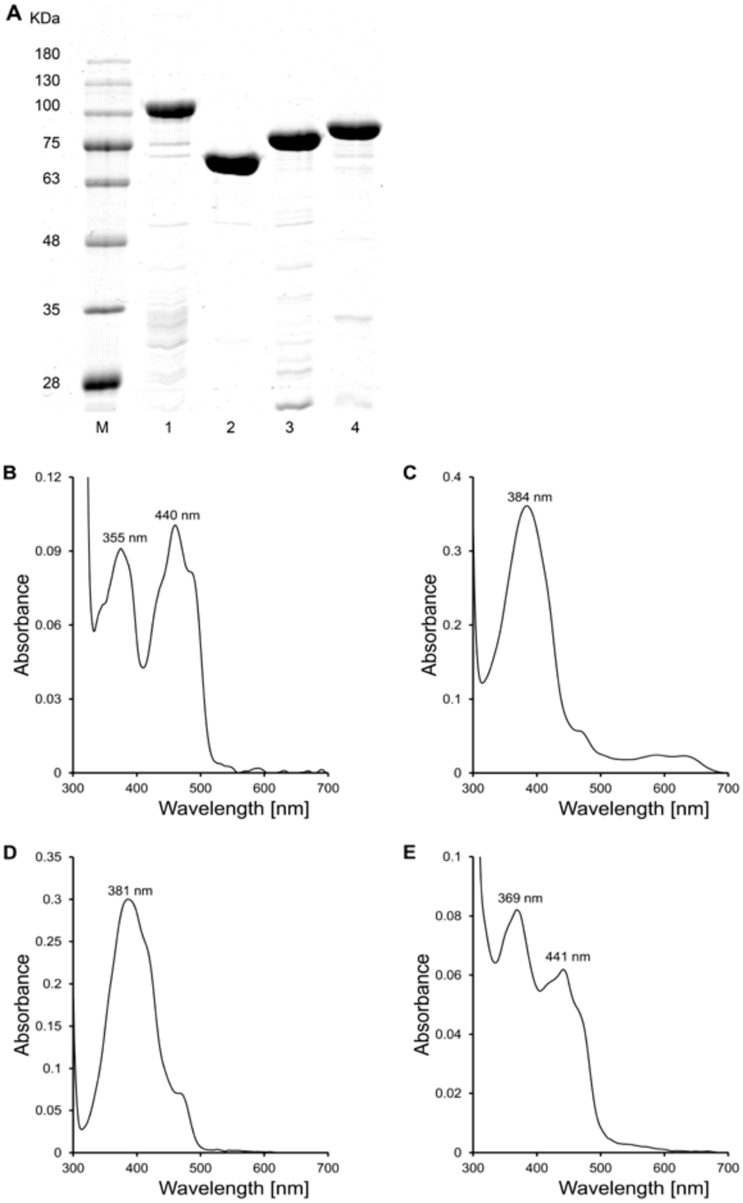

Fig. S2. Purification and absorption spectra of *U. maydis* CPF members. (A) Coomassie-stained gel showing *E. coli*-expressed CPF members of *U. maydis* after Ni-NTA and heparin column purification. In each lane 5 µg of protein were loaded. The calculated molecular masses of CPF members are given in brackets. Lane 1: Cry1, um01131 (103 kDa); lane 2: Cry2, um05917 (76 kDa); lane 3: Phr2, um02144 (82 kDa); lane 4: Phr1, um06079 (88 kDa). (B-E) Absorption spectra of (B) Cry1; (C) Cry2; (D) Phr1 and (E) Phr2. Protein samples were kept in darkness before taking the spectra.

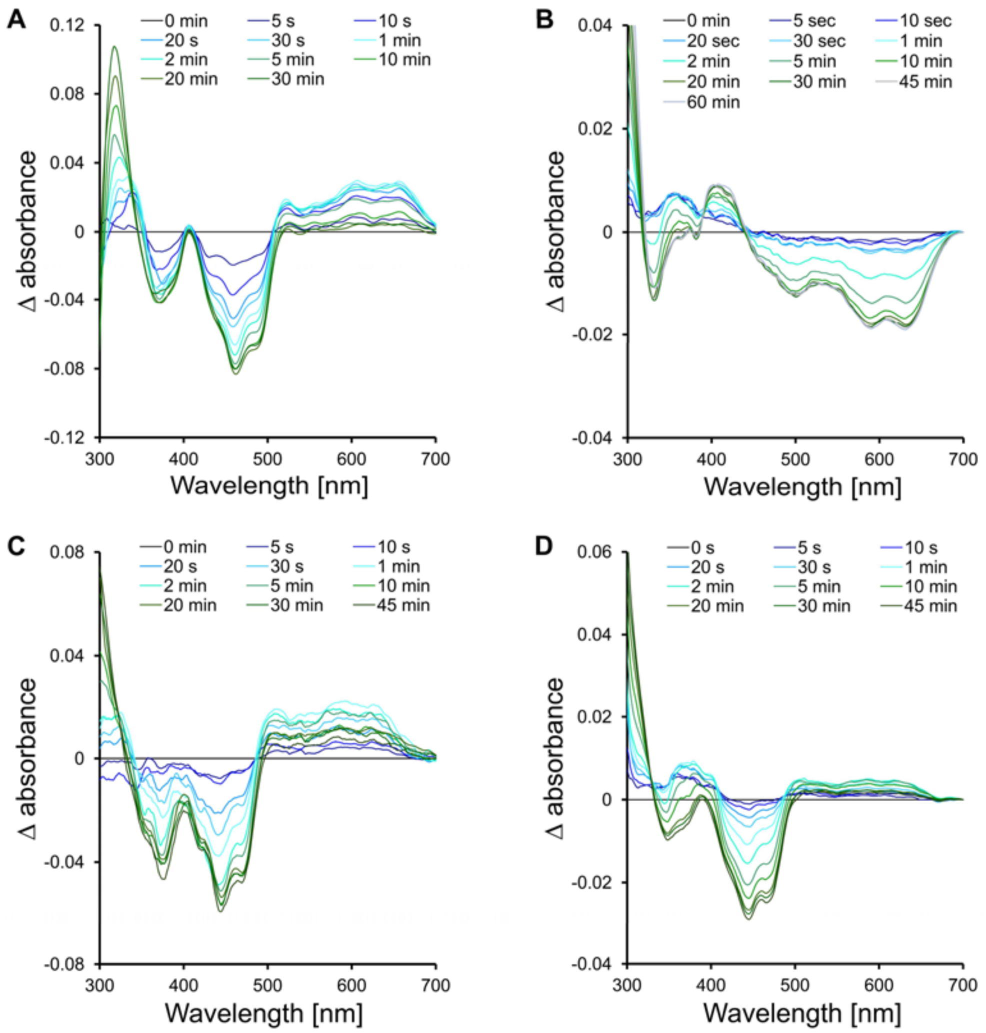

Fig. S3. Light-dark difference spectra of *U. maydis* CPF members. (A) Cry1 treated with blue light (450 nm, 50 µmol·m^{-2}·s^{-1}). (B) Cry2 treated with blue light (450 nm, 64 µmol·m^{-2}·s^{-1}). (C) Phr1 treated with blue light (450 nm, 100 µmol·m^{-2}·s^{-1}). (D) Phr2 treated with blue light (439 nm, 38 µmol·m^{-2}·s^{-1}). Irradiation times are indicated.

Tab. S1. *U. maydis* strains used in this study.

Strain	Relevant genotype	Reference
FB1	*a1 b1*	Banuett and Herskowitz 1989
FB1 Δ*wco1*	*a1 b1* Δ*wco1*	this study
FB1 Δ*wco2*	*a1 b1* Δ*wco2*	Heimel *et al.* 2010
FB1 Δ*phr1*	*a1 b1* Δ*phr1*	this study
FB1 Δ*phr2*	*a1 b1* Δ*phr2*	this study
FB1 Δ*cry1*	*a1 b1* Δ*cry1*	this study
FB1 Δ*cry2*	*a1 b1* Δ*cry2*	this study
FB1 Δ*phr1*/Δ*phr2*	*a1 b1* Δ*phr1;* Δ*phr2*	this study
FB1 Δ*cry1*/Δ*cry2*	*a1 b1* Δ*cry1;* Δ*cry2*	this study
FB1 GFP-Wco1	*a1 b1 ip*R[P_{otef}: *egfp-wco1*]*ip*S	this study
FB1 mCherry-Wco2	*a1 b1 ip*R[P_{otef}: *mcherry-wco2*]*ip*S	this study

References

Banuett, F., and I. Herskowitz. 1989. Different alleles of *Ustilago maydis* are necessary for maintenance of filamentous growth but not for meiosis. Proc. Natl. Acad. Sci. USA 86:5878-5882.

Heimel, K., Scherer, M., Schuler, D., and J. Kämper. 2010. The *Ustilago maydis* Clp1 protein orchestrates pheromone and b-dependent signaling pathways to coordinate the cell cycle and pathogenic development. Plant Cell 22:2908-2922.

Tab. S2. Primers used for creating *U. maydis* deletion strains.

Name	Sequence (5'→3')	Description
MG 410	atataatattCGCCTCTCTCTAGGCAATCTACC	Construction of *Δwco1;* left flank fw
MG 411	atatggccatctaggccTTGAAGGGGCAATCACTAATTGC	Construction of *Δwco1;* left flank rv
MG 412	atatggcctgagtggccAGCGCGTACCAAGCTCTCTCTCC	Construction of *Δwco1;* right flank fw
MG 413	atataatattCTTATTACTTACTACCACAGTGC	Construction of *Δwco1;* right flank rv
MG946	ATATAATATTAGTAAGGCGGAGCCAAGGAGG	Construction of *Δcry1;* left flank fw
MG947	ATATGGCCATCTAGGCCAGGATGATGAGCGGCTTCTT	Construction of *Δcry1;* left flank rv
MG948	ATATGGCCTGAGTGGCCCCATGGTCACACATGTAGTAATAAT	Construction of *Δcry1;* right flank fw
MG949	ATATAATATTGGCAGACGAGGAAACATGCG	Construction of *Δcry1;* right flank rv
MG950	ATATAATATTTACGCCAGCGTAAGGCATGAATCGGAAAAC	Construction of *Δcry2;* left flank fw
MG951	ATATGGCCATCTAGGCCACAAAAGCGTCAAGCACATA	Construction of *Δcry2;* left flank rv
MG952	ATATGGCCTGAGTGGCCGCAACAACAGATCTCCCATA	Construction of *Δcry2;* right flank fw
MG953	ATATAATATTTGTCCGGGTTCAGGTGATCGCCACGG	Construction of *Δcry2;* right flank rv
MG 416	atataatattCACCACCCGCGTCCGTCTCTACG	Construction of *Δphr1;* left flank fw
MG 417	atatggccatctaggccTAGACGCAAGTAACTTAAACACC	Construction of *Δphr1;* left flank rv
MG 418	atatggcctgagtggccTGTGAGGACTGTACGGCTGGTAG	Construction of *Δphr1;* right flank fw
MG 419	atataatattTAGCCTTGCCCAACGGCTGACC	Construction of *Δphr1;* right flank rv
MG 422	atataatattCAGAGCAGTACCTTGGCCTGC	Construction of *Δphr2;* left flank fw
MG 423	atatggccatctaggccGATTGTTTGAAAATCTGTAGTCC	Construction of *Δphr2;* left flank rv
MG 424	atatggcctgagtggccGGATAATGGTAGAGAGTTTGC	Construction of *Δphr2;* right flank fw
MG 425	atataatattAGTGGAGCATTGTTCGTTAGG	Construction of *Δphr2;* right flank rv

Tab. S3. Primers used for qRT-PCR and gene cloning.

Name	Sequence 5’ to 3’
qRT-PCR *cyclophilin* (internal standard) fwd	GAACTTCAAGCTCTCGCAC
qRT-PCR *cyclophilin* (internal standard) rev	TCTCGATAGCCTTGACGATG
qRT-PCR *cry1* fwd	CTTCATGTCAAACCGTGGTC
qRT-PCR *cry1* rev	TGAGATGCGACTGGAAGAAC
qRT-PCR *cry2* fwd	TTGACCGATCTGACTCTTGC
qRT-PCR *cry2* rev	GCCGAATTTGGTCGAGTAGT
qRT-PCR *phr1* fwd	CTCACCAAACATCTCCTCCAC
qRT-PCR *phr1* rev	TCGCATTTTTCGCTCTGGC
qRT-PCR *phr2* fwd	GTCAGCTGCTATGACACCGT
qRT-PCR *phr2* rev	TACAAATGTTGGCGAGCTTC
qRT-PCR *phy1* fwd	CTCAACAGGCTCCTCCTCTC
qRT-PCR *phy1* rev	GCGAGATGCGATTTACTCCAA
qRT-PCR *wco1* fwd	GCTGTCCTAGGTGCAGATGA
qRT-PCR *wco1* rev	AGCACGTAGATGAGGTCGTG
qRT-PCR *wco2* fwd	GTCCTAGCTCCAACGGTAGC
qRT-PCR *wco2* rev	TTGTTGTGGAAGAGCAAAGG
qRT-PCR *ops1* fwd	ACTCTTCGCTCGTTCTCACC
qRT-PCR *ops1* rev	CCTCGTAGTCTGAAAACTTGCC
qRT-PCR *ops2* fwd	ACCTTCGATCCAACATGCC
qRT-PCR *ops2* rev	AGCCTTTTTCTCTGCCCTCC
qRT-PCR *ops3* fwd	CTCACCAAACATCTCCTCCAC
qRT-PCR *ops3* rev	TCGCATTTTTCGCTCTGGC
qRT-PCR *blf1* fwd	CTCAACAGGCTCCTCCTCTC
qRT-PCR *blf1* rev	GCGAGATGCGATTTACTCCAA
Cloning of *Δwco1* LF fwd	atataat'attCGCCTCTCTCTAGGCAATCTACC
Cloning of *Δwco1* LF rev	atatggccatct'aggccTTGAAGGGGCAATCACTAATTGC
Cloning of *Δwco1* RF fwd	atatggcctgag'tggccAGCGCGTACCAAGCTCTCTCTCC
Cloning of *Δwco1* RF rev	atataat'attCTTATTACTTACTACCACAGTGC
Cloning of P_{otef}*:gfp:wco1* fwd	tacga'cgcgtaACGGATACTTTCGATTTCGACGCT
Cloning of P_{otef}*:gfp:wco1* rev	aacggc'ggccgcAGGCTTCGGCCGAGCCAGAG
Cloning of *Δwco2* LF fwd	gacaat'attGAGAGTAACTGATACTCTCGAC
Cloning of *Δwco2* LF rev	atatggccatct'aggccGTCTCGAGTTGAAGTGCAGT
Cloning of *Δwco2* RF fwd	atatggcctgag'tggccGACCTCATCATCCTGGCG
Cloning of *Δwco2* RF rev	gacaat'attGATATGACTCTTTCATACAGCCA
Cloning of P_{otef}*:mCherry:wco2* fwd	tacga'cgcgtaCAGGGTCAGGGTTGGTCTAGC
Cloning of P_{otef}*:mCherry:wco2* rev	aacggc'ggccgcTCACATGTGACCGCCGCCCA
Cloning of *Δphr1* LF fwd	atataat'attCACCACCCGCGTCCGTCTCTACG
Cloning of *Δphr1* LF rev	atatggccatct'aggccTAGACGCAAGTAACTTAAACACC
Cloning of *Δphr1* RF fwd	atatggcctgag'tggccTGTGAGGACTGTACGGCTGGTAG
Cloning of *Δphr1* RF rev	atataat'attTAGCCTTGCCCAACGGCTGACC
Cloning of *Δphr2* LF fwd	atataat'attCAGAGCAGTACCTTGGCCTGC
Cloning of *Δphr2* LF rev	atatggccatct'aggccGATTGTTTGAAAATCTGTAGTCC
Cloning of *Δphr2* RF fwd	atatggcctgag'tggccGGATAATGGTAGAGAGTTTGC
Cloning of *Δphr2* RF rev	atataat'attAGTGGAGCATTGTTCGTTAGG
Cloning of *Δcry1* LF fwd	atataat'attAGTAAGGCGGAGCCAAGGAGG
Cloning of *Δcry1* LF rev	atatggccatct'aggccAGGATGATGAGCGGCTTCTT
Cloning of *Δcry1* RF fwd	atatggcctgag'tggccCCATGGTCACACATGTAGTAATAAT
Cloning of *Δcry1* RF rev	atataat'attGGCAGACGAGGAAACATGCG
Cloning of *Δcry2* LF fwd	atataat'attTACGCCAGCGTAAGGCATGAATCGGAAAAC
Cloning of *Δcry2* LF rev	atatggccatct'aggccACAAAAGCGTCAAGCACATA
Cloning of *Δcry2* RF fwd	atatggcctgag'tggccGCAACAACAGATCTCCCATA
Cloning of *Δcry2* RF rev	atataat'attTGTCCGGGTTCAGGTGATCGCCACGG
Y2H cloning of *wco1* fwd	aattg'aattcTCAGGCTTCGGCCGAGCCAG
Y2H cloning of *wco1* rev	gacaat'attgAGAGTAACTGATACTCTCGAC
Y2H cloning of *wco2* fwd	aattg'aattcACATGTGACCGCCGCCCA
Y2H cloning of *wco2* rev	ccggccc'ggggCAGGGTCAGGGTTGGTCTAGC

Tab. S4. *E. coli* strains used in this study.

strain	genotype
BL21 (DE3) pLysS	F^- *ompT* *hsdS*$_B$(r_B^- m_B^-) *gal dcm* lon λ (DE3) pLysS (Cam^R)
Rosetta2 (DE3) pLysS	F^- *ompT* *hsdS*$_B$(r_B^- m_B^-) *gal dcm* λ (DE3) pLysS RARE2 (Cam^R)
Arctic Express (DE3)	*E. coli* B F– *ompT* *hsdS*(r_B^- m_B^-) *dcm*$^+$ λ (D3) Tet^R *gal endA* Hte [*cpn10 cpn60* $Gent^R$]
TOP10	*F- mcrA Δ(mrr-hsdRMS-mcrBC) Φ80lacZΔM15 Δ lacX74 recA1 araD139 Δ(araleu)7697 galU galK rpsL (*Str^R*) endA1 nupG*
DH5α	*F- Φ80lacZΔM15 Δ(lacZYA-argF) U169 recA1 endA1 hsdR17 (*r_K^-, m_K^+*) phoA supE44 λ*$^-$ *thi-1 gyrA96 relA1*

Tab. S5. Primers used in this study for *E. coli* expression constructs.

Name	Sequence 5' to 3'
cry1 fwd	GGTACCAGAAGGTAGCAG CAGCAGCGATAG C
cry1 rev	GAGCTCCCTGTTTGCGTTCAT AATGACGCT
cry2 fwd	ATACGTCGACACACGCAA CATCCTCATCGC
cry2 rev	ATACGAATGCGGCCGCTGA CTGCTGG
phr1 fwd	CATGCCATGGCGCCAAA ACGCAAAGGAGCCG
phr1 rev	AATATGCGGCCGCTGGTCACC AGGATTCTTGTACC
phr2 fwd	GGTACCGTCGAAGCACGTC CGCGTCTTATAC
phr2 rev	GCG GCCGCTTCTTTCTTTTTG CTTGTACCCTCTAC

Tab. S6. Probes used for *in vitro* repair assays.

Name	Sequence 5’ to 3’
oligo LAMRA	AAAATGCTGGATGTCGAGGTGTAAT<>TAATGTGGAGCTGTAGGTCGTAAAA
oligo CT	CGACCTACAGCTCCACATTAATTACACCTCGACATCCAGC
oligo $dT_{(18)}$	TTTTTTTTTTTTTTTTTT
